# p53 Specifically Binds Triplex DNA *In Vitro* and in Cells

**DOI:** 10.1371/journal.pone.0167439

**Published:** 2016-12-01

**Authors:** Marie Brázdová, Vlastimil Tichý, Robert Helma, Pavla Bažantová, Alena Polášková, Aneta Krejčí, Marek Petr, Lucie Navrátilová, Olga Tichá, Karel Nejedlý, Martin L. Bennink, Vinod Subramaniam, Zuzana Bábková, Tomáš Martínek, Matej Lexa, Matej Adámik

**Affiliations:** 1 Department of Biophysical Chemistry and Molecular Oncology, Institute of Biophysics, Academy of Sciences of the Czech Republic v.v.i., Brno, Czech Republic; 2 Department of Molecular Biology and Pharmaceutical Biotechnology, Faculty of Pharmacy, University of Veterinary and Pharmaceutical Sciences Brno, Brno, Czech Republic; 3 Biophysical Engineering Group, Faculty of Science and Technology, University of Twente, Enschede, The Netherlands; 4 Department of Computer Systems, Faculty of Information Technology, Brno University of Technology, Brno, Czech Republic; 5 Department of Information Technologies, Faculty of Informatics, Masaryk University, Brno, Czech Republic; Saint Louis University, UNITED STATES

## Abstract

Triplex DNA is implicated in a wide range of biological activities, including regulation of gene expression and genomic instability leading to cancer. The tumor suppressor p53 is a central regulator of cell fate in response to different type of insults. Sequence and structure specific modes of DNA recognition are core attributes of the p53 protein. The focus of this work is the structure-specific binding of p53 to DNA containing triplex-forming sequences *in vitr*o and in cells and the effect on p53-driven transcription. This is the first DNA binding study of full-length p53 and its deletion variants to both intermolecular and intramolecular T.A.*T* triplexes. We demonstrate that the interaction of p53 with intermolecular T.A.*T* triplex is comparable to the recognition of CTG-hairpin non-B DNA structure. Using deletion mutants we determined the C-terminal DNA binding domain of p53 to be crucial for triplex recognition. Furthermore, strong p53 recognition of intramolecular T.A.*T* triplexes (H-DNA), stabilized by negative superhelicity in plasmid DNA, was detected by competition and immunoprecipitation experiments, and visualized by AFM. Moreover, chromatin immunoprecipitation revealed p53 binding T.A.*T* forming sequence *in vivo*. Enhanced reporter transactivation by p53 on insertion of triplex forming sequence into plasmid with p53 consensus sequence was observed by luciferase reporter assays. *In-silico* scan of human regulatory regions for the simultaneous presence of both consensus sequence and T.A.*T* motifs identified a set of candidate p53 target genes and p53-dependent activation of several of them (*ABCG5*, *ENOX1*, *INSR*, *MCC*, *NFAT5*) was confirmed by RT-qPCR. Our results show that T.A.*T* triplex comprises a new class of p53 binding sites targeted by p53 in a DNA structure-dependent mode *in vitro* and in cells. The contribution of p53 DNA structure-dependent binding to the regulation of transcription is discussed.

## Introduction

Tumor suppressor p53 contains two DNA binding domains. The central (core) domain (amino acids ~100 to ~300) is evolutionarily highly conserved and is essential for p53 sequence-specific binding to promoters of p53 target genes that take part in cell cycle regulation, apoptosis and DNA repair [1]. The p53 consensus sequence (CON) has been originally defined as two copies of the sequence 5´-PuPuPuC(A/T)(T/A)GPyPyPy-3´ separated by 0–13 bp [2]. The core domain also binds in non-sequence-specific manner to single- and double-stranded DNA, preferentially interacting with internal regions of single-stranded (ss) DNA [3], three-stranded DNA substrates mimicking early recombination intermediates [4], insertion/deletion mismatches [5] and DNA cruciform stabilized by DNA superhelicity [6]. The C-terminal part of the protein contains a flexible linker (amino acids ~300 to ~325), a tetramerization domain (amino acids ~325–356) and a basic C-terminal DNA binding domain (CTDBD, aa 363–382). The ability of the C-terminus to bind single-stranded gaps in double-stranded (ds) DNA [7], cisplatin-modified DNA [8], hemicatenated DNA loops [9] and superhelical DNA (scDNA [10, 11]) has been described. There is a growing amount of data suggesting that p53 interactions with different DNA targets represent a complex network involving contributions from both DNA binding domains reviewed in [12]. Recently, we have shown that the human telomeric G-quadruplexes are recognized by full length p53 protein and both DNA-binding domains take part in this interaction [13].

The triple-helical (triplex) DNA adopts a structure characterized by a third pyrimidine-rich or purine-rich DNA strand located within the major groove of a homopurine/homopyrimidine stretch of duplex DNA [14–16]. Stable interaction of the third strand is achieved through either specific Hoogsteen or reverse Hoogsteen hydrogen bonding with the homopurine strand of the duplex. Preferred base triplets include T.A.*T* and C.G.*C* in the pyrimidine motif and C.G.*G* and T.A.*A* in the purine motif. Triplexes can be either intermolecular, where the third strand originates from a separate DNA molecule, or intramolecular (named also H-DNA), where the third strand originates from the same DNA molecule as its duplex acceptor [15, 16]. Naturally occurring sequences capable of forming intramolecular triplex are found in human genome as frequently as 1 in every 50000 bp [17] and are enriched in introns and promoters [18, 19]. Intramolecular triplexes are postulated to occur *in vivo* under suitable conditions (such as sufficiently high negative superhelical stress) and their involvement has been implicated in several cellular processes, including transcription, replication and recombination [15, 16]. The triplex target sequence for formation of intermolecular DNA triplexes is even more abundant, on average one unique triplex target sequence every 1366 bases [20]. Intermolecular triplexes are widely recognized as potential tools for different genetic manipulations including gene regulation and mutagenesis [21, 22]. So far, only a few proteins recognizing triplexes of pyrimidine type are known [23–26]. The importance of triplex DNA for the occurrence of some breakpoint hotspots in cancer has also been hypothesized [27]. Despite the correlation between genomic instability and formation of triplex DNA, the function of proteins that recognize these structures is still poorly understood. Several DNA repair proteins have been shown to bind triplex DNA [23].

Negative DNA superhelicity is necessary for the formation of intramolecular triplex DNA (H-DNA) and other non-B DNA structures *in vivo* [28]. Observations from our laboratory [11, 29, 30], as well as of others [12, 31] have revealed a clear relationship between the topology of recognized DNA and p53. Both wild-type p53 and mutant p53 proteins have considerable potential to recognize non-B DNA structures. In particular, formation of stem-loop, hairpin or cruciform structures affects p53-DNA interactions [12, 30–33].

In this study, we have analyzed for the first time the interaction of the full-length p53 and its deletion variants to DNA containing triplex-forming sequences *in vitr*o and in cells. We show that p53 protein possessing intact C-terminus exhibits high affinity to intermolecular and intramolecular T.A.*T* triplex DNA. *In-silico* analysis of human promoters for simultaneous presence of consensus sequence and T.A.*T* motifs identified a set of candidate p53 target genes. Possible contribution of DNA triplex-dependent binding of p53 for regulation of their transcription is discussed.

## Material and Methods

### Oligonucleotides

The sequences of oligonucleotides used in this study are presented in S1 Table, oligonucleotides were synthesized by VWS (Vienna, Austria). Duplex and triplex probes were prepared as previously described [25]. Briefly, intermolecular T.A.*T* triplex (oligo(dT)_50_.oligo(dA)_50_.oligo(dT)_50_) was formed by standard annealing of (dT)_50_ to labeled (dA)_50_ and titration of duplex with (dT)_50_ to molar excess (3–5×) in presence of Mg^2+^ ions in triplex forming buffer (5 mM Tris-HCl, pH 8, 1 mM MgCl_2_, 300 mM NaCl) at 37°C for 60 min. CTG_hairpin_ and TA_hairpin_ were prepared as described in [32] with labeled lock oligonucleotide (S1 Table).

### Recombinant plasmids

Plasmids encoding human p53 proteins pT7-7wtp53 (full length wild type p53,p53, aa 1–393), pET-p53CD (p53CD, aa 94–312), pGEX-2TKp53CT (GST-p53CT, aa 320–393), pGEX-2TKp53T (GST-p53T, aa 363–393) and pGEX-4Tp53CD (GST-p53CD, aa 94–312) were described in [10, 29]. Plasmids with T.A.*T* triplex forming sequences (pBA50 and pPA50) were prepared by cloning of (dT)_50_.(dA)_50_ into the *Eco*RV site of pBluescript SK II- (pBSK, Stratagene) and pPGM1 [34] (S1 Table). Similarly, plasmids for cruciform formation (pBAT34, pPAT34) were prepared by cloning (dAdT)_34_ sequences to the same plasmids, for details see S1 Table. Plasmid pA69 with (dT)_69_.(dA)_69_ (on pUC19 basis [35]) and pUC19 control plasmid were used. Nonspecific competitor (pBSK/*Sma*I) was prepared by *Sma*I restriction enzyme (Takara, Japan) cleavage of pBSK. Plasmids for luciferase reporter assay (pGL3-BSK, pGL3-P1, pGL3-BA50, pGL3-PA50, pGL3-PA20_,_ S1 Table) were constructed by cloning fragments from pBSK derivatives into the *Sma*I/*Xho*I site of the pGL3-promoter (Invitrogen). All plasmids were isolated from bacterial strain TOP10 (Stratagene) and verified by sequencing.

### p53 recombinant proteins purification

Full length p53 and isolated DNA binding domains p53CD, p53CT, and p53T (with or without GST tag) were purified according to a protocol described previously [10, 29]. The purity and appropriate size of each protein were analyzed by Coomassie blue staining of 12.5% SDS-PAGE gels (S1A Fig), using bovine serum albumin as a standard.

### EMSA in polyacrylamide gels

^32^P-radiolabeled oligonucleotide probes (1 pmol) were mixed with p53 proteins and incubated in binding buffer (5 mM Tris-HCl, pH 8, 1 mM MgCl_2_, 0.01% Triton X-100 and 50 mM KCl) in the presence of 5–50 ng pBSK/*Sma*I competitor DNA for 30 min on ice or at 25°C to reach equilibrium. Samples were loaded onto a 4–5% polyacrylamide gel containing 0.5× TB buffer with 2 mM MgCl_2_. After 1–3 h electrophoresis (at 4–6 V/cm^2^) the gels were dried and DNA was detected by autoradiography using Typhoon FLA 9000 (GE Healthcare). Polyclonal rabbit CM1 and mouse monoclonal (DO1 (aa 20–25), Bp53-10.1 (aa 375–379), PAb421 (aa 371–380) and ICA9 (aa 388–393)) antibodies, kindly provided by Dr. B. Vojtesek, were used in supershift and IP experiments.

### ELISA

96-well Immuno Plates (SPL LIFE SCIENCES) were streptavidin (PROSPEC) coated and blocked for unspecific binding by BSA (Sigma). Biotinylated oligonucleotides (0.5 pmol) were bound to the plate and then pre-incubated protein-primary antibody mixes (in 2/1 Ab/protein molar ratio) were added. Secondary HRP-labeled antibody was incubated on ELISA plate for 30 min, washed and then TMB substrate was added. Absorbance was measured at 370 nm on Synergy H1 (BioTek) and evaluated in GraphPad Prism using hyperbolic or Hill equation fittings. All wash and incubation steps were done in the presence of 2 mM MgCl_2_ in 1× PBS. Kd were obtained from at least three independent measurements. Details of the procedures are described in [13].

### EMSA in agarose gels

scDNAs (200 ng pBSK, pPGM1, pBA50, pPA50) were preincubated in triplex-forming buffer at 37°C for 30 min. scDNAs were mixed with p53 proteins in p53 tetramer/DNA molar ratios 0.25–5 and incubated in binding buffer (5 mM Tris-HCl, pH 8, 1 mM MgCl_2_, 0.01% Triton X-100 and 50 mM KCl) for 30 min either on ice or 25°C to reach equilibrium. Samples were loaded onto a 1% agarose gel containing 0.33× Tris-borate-EDTA (TBE) buffer. After 5 h electrophoresis (at 4–6 V/cm^2^) agarose gels were stained with ethidium bromide (EtBr) and photographed. Intensities of bands of free DNA substrates were quantified using ImageQuant software. Graphs show the evaluation of p53-DNA binding as the dependence of % of bound DNA on the amount of p53 proteins (expressed by molar ratio p53/DNA), more details in [29]. Mean values of three independent experiments were plotted in the graph.

### Immunoprecipitation assay

The DO1-p53-DNA complexes were prepared by mixing the DO1 antibody (400 ng) with the purified protein (50 ng) in binding buffer followed by 20 min incubation on ice. Then, 200 ng of scDNA (preincubated in triplex-forming buffer) and the same amount of linDNA (pBSK/*Sma*I) were mixed with the given complexes and incubated in the binding buffer for 30 min on ice. Magnetic beads (12 μl of suspension per sample) coated with protein G (MBG, Dynal/Invitrogen) were added to DO1-p53-DNA complexes after washing in binding buffer and incubated with the beads for 30 min at 10°C. Finally, after washing in binding buffer with increased salt concentration (1× 50 mM, 2× 50–600 mM, 1× 50 mM), DNA was released from the beads by heating at 65°C in 15 μl of 1.0% SDS for 5 min and analyzed by agarose gel electrophoresis, more details in [29]. Intensities of bands of bound DNA substrates were quantified using ImageQuant software. Graphs show the evaluation of p53-DNA binding as the dependence of % of bound DNA on the concentration of KCl. Mean values of three independent experiments were plotted in the graph.

### Human cell lines, transfections and luciferase assays

Human breast adenocarcinoma MCF7 (HTB-22, ATCC), human non-small cell lung carcinoma line H1299 (NCI-H1299, ATCC) and H1299-wtp53 cells (Tet-On system, [36]) were grown in DMEM medium supplemented with 5% FBS and penicillin/streptomycin (Gibco). All cultures were incubated at 37°C with 5% CO_2_. The luciferase reporter constructs (S1 Table) containing CON and/or (dA)_50_ or (dA)_20_ sequences were used for luciferase assay as described in [29]. pRL-SV40 was used as a transfection efficiency control. 200 ng of reporter construct was transfected in triplicates. Luciferase activity was measured in a plate reader luminometer IMMUNOTECH LMT01 (Beckmann) with Dual Luciferase Assay System (Promega). For each construct, relative luciferase activity is defined as the mean value of the Firefly luciferase/Renilla luciferase activity ratios obtained from at least three independent experiments.

### RT-qPCR

Total RNA was isolated using NucleoSpin RNA II (Macherey-Nagel) and 2 μg of RNA was subsequently reverse transcribed into cDNA by applying High Capacity RT kit (Applied Biosystems). qPCR was performed using EvaGreen (Solis Biodyne) fluorescent dye in the standard program (15 min 95°C; 15 s 95°C, 30 s 60°C, 20 s 72°C, 10 s 74°C; 50 cycles) running in RotorGene 6000 (Corbett Research). RT-qPCR reactions for each sample were measured in triplicates. GAPDH was used as reference gene. Absolute quantification was performed using standard curve method. Data were then normalized to GAPDH. The housekeeping genes (HPRT1, GAPDH) were used as endogenous controls. Relative quantification of transcript levels with respect to the calibrator (H1299 with empty vector, MCF7 siRNA control, MCF7) was done based on 2^-ΔΔCT^ algorithm. All reactions were carried out in biological triplicates. The primer sequences used are listed in S1 Table.

### Immunoblotting

H1299 and Hwtp53 (expressing wtp53, induced with 1 μg/ml tetracycline for 24 hours) cells were harvested from 10 cm plates and lysed with 1× PLB (Promega), followed by the sonication of cells (Bandelin Sonopuls). Samples (100 μg of total protein) were analyzed on 12.5% SDS-PAGE gels and proteins were detected by the following primary antibodies: DO1 (anti-p53, kindly provided by B. Vojtesek), anti-CDKN1A (Millipore), anti-β-Actin (Sigma), anti-BAX (Sigma), anti-NAT10 (ThermoScientific).

### Chromatin immunoprecipitation

Human breast adenocarcinoma MCF7 treated for 4 hours with nutlin-3 (5 μM) or doxorubicine (1 μM) were subjected to chromatin immunoprecipitation (ChIP) assays as previously described [29] with the following modifications: the cell sonication was limited to 4 kJ (Bandelin Sonopuls). Purified monoclonal DO1 antibody and IgG (negative control) were incubated overnight with diluted chromatin and immunoprecipitations were performed with protein G-magnetic beads (Invitrogen). The PCR was performed using the primers targeting expected p53 binding site (S1 Table). In other type of ChIP experiment was performed with H1299 cells transfected with plasmids pGL3-PGM1 and pGL3-BA50 (2 μg) and p53 expression vector (pCDNA3.1; 1 μg), after 16 hours cells were subjected to chromatin immunoprecipitation (ChIP) assays. The PCR was performed using the primers targeting expected p53 binding site in pGL3 vector or native promoter sequence see in S1 Table. For quantitative analysis, PCR was carried out for 25 or 30 cycles.

### *In-silico* analysis of promoter regions

Human regulatory sequences were obtained using Table Browser [37] and saved as a FASTA-formatted file of -5000bp to +2000bp regions around each RefSeq TSS. The CON binding sites were identified as closely (<21bp) located pairs of sequence motifs with a maximum of 1 mismatch. The set of identified p53CON sites was expanded to include all full-length grade 3–5 sites identified by p53retriever R/Bioconductor package [38], which largely overlapped the original set. The identification of potential triplex-forming sequences was carried out using the R/bioconductor program triplex-1.8.0 [19], using the default scoring scheme of the software tested in our previous work on human sequences [39]. To check for possible common functions of the identified proteins, we performed a network enrichment analysis using the STRING database tool [40] and gProfiler [41].

### *In-silico* candidate gene transcription screening

Candidate gene transcription was checked in publicly available microarray and sequencing datasets from experiments involving p53-transformed cells originally lacking active p53 or experiments were p53 was activated by nutlin-3, 5-fluoruracil or doxorubicin (SRP043273, SRP022871, E-GEOD-30753, E-GEOD-50650, E-GEOD-8660, E-MEXP-2556 [42]). We obtained expression data from tables available from the iRAP pipeline [43], deposited by authors to Array Express [44] or calculated from the available data using the ArrayExpress R/Bioconductor package [45]. Raw expression values were normalized relative to GAPDH housekeeping gene and averaged, where replicates were available.

### Atomic Force Microscopy (AFM)

AFM measurements were carried out on MultiMode 8 system (Bruker) with NanoScope 8.15 software or on a custom-built AFM system [46]. 50 A silicon nitride MSCT probe, cantilever F (k = 0.5 N/m, Bruker, Santa Barbara, CA, USA), was used with a free amplitude between 1 and 2 nm (amplitude set point between 0.8 and 1.5 nm, 80–90% of the free amplitude). Plasmids were incubated in binding buffer at 37°C for at least 30 min. For p53-DNA complex images, plasmids were mixed with p53 proteins in p53 tetramer/DNA molar ratio 5/1 and incubated on ice for 20 min. Sample containing 2 ng of plasmid DNA was diluted in 4 mM HEPES pH 7.6, 5 mM MgCl_2_, 5 mM KCl buffer and placed on freshly cleaved mica V4 surface, incubated for 2 min, washed with distilled water and dried with a stream of compressed air.

## Results

### Full length p53 binding to intermolecular T.A.*T* triplex is comparable with CTG hairpin non-B DNA structure recognition

**Fig 1:**
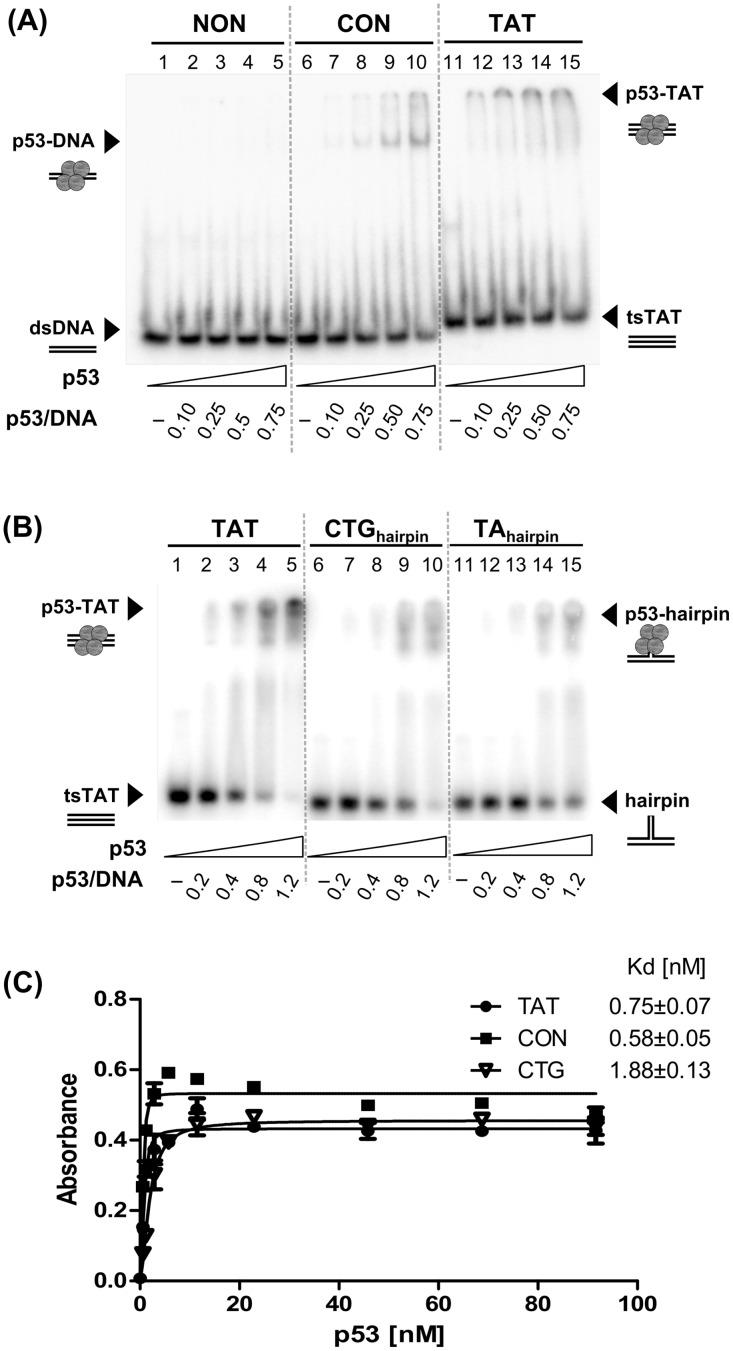
Full length p53 binds strongly to T.A.*T* triplex DNA. **(A)** Full length p53 was incubated with 1 pmol of ^32^P-labeled 50-mer oligonucleotides: nonspecific dsDNA (NON, lanes 1–5), p53 specific dsDNA with CON (CON, lanes 6–10) and (dT)_50_.(dA)_50_.(dT)_50_ triplex (T.A.*T* triplex, lanes 11–15) in presence of 50 ng pBSK/*Sma*I. Molar ratios of p53 tetramer/DNA ranged between 0.1 and 0.75. The samples were loaded onto 5% 0.5 × TBM (2 mM MgCl_2_) polyacrylamide gel and electrophoresis was performed for 0.45 h. **(B)** Full length p53 was incubated with 1 pmol of ^32^P-labeled (dT)_50_.(dA)_50_.(dT)_50_ triplex (T.A.*T* triplex, lanes 1–5), CTG hairpin (lanes 6–10) and TA hairpin (lanes 11–15) oligonucleotides in presence of 50 ng pBSK/*Sma*I. Molar ratios of p53 tetramer/DNA ranged between 0.2 and 1.2. The samples were loaded onto 5% 0.5 × TBM (2 mM MgCl_2_) polyacrylamide gel and electrophoresis was performed for 0.45 h. **(C)** p53 binding to biotinylated oligonucleotides by ELISA. p53 binding curves for the TAT, CON and CTG oligonucleotides are shown, and the dissociation constants (Kd) are indicated.

Wild type p53 protein is well-known as a non-B DNA structure binder but its interaction with triplex DNA has not been studied yet. We examined p53 binding to pyrimidine type of triplex DNA formed by homoadenine and homothymine oligonucleotides. Intermolecular T.A.*T* triplex was formed in neutral pH in the presence of Mg^2+^ ions [25]. Binding of full-length wild type p53 (p53, Fig 1A) to T.A.*T* triplex was examined by EMSA in the presence of Mg^2+^ ions. Increasing amounts of p53 (50–500 ng, Fig 1A) were bound to 50 bp long random sequence (NON, lanes 2–5), p53 consensus sequence (CON, lanes 7–10) and T.A.*T* triplex (TAT, lanes 12–15). We observed small differences in p53 binding to T.A.*T* triplex (Fig 1A, TAT, lanes 12–15) and to CON (lanes 7–10). In comparison with CTG_hairpin_ (Fig 1B, lanes 7–10) and TA_hairpin_ (Fig 1B, lanes 12–15), the T.A.*T* triplex (Fig 1B, lanes 2–5) was bound by p53 stronger. Considerably weak binding was observed to NON (Fig 1A, lanes 2–5). Detailed titration of p53 protein to T.A.*T* triplex and CON substrates (S1 Fig) mapped the differences between recognition of both substrates.

To better characterize the differences in p53 binding to T.A.*T* triplex in comparison with CON and CTG_hairpin_, we employed an enzyme-linked immunosorbent assay (ELISA) with a set of biotinylated target oligonucleotides CON, TAT and CTG_hairpin_ as recently described for p53-quadruplex DNA binding [13]. Incubation of the immobilized target oligonucleotides with a range of p53 protein (0.1–90 nM) was followed by quantitation using DO1 antibody. Using this system, we demonstrated that p53 binds to T.A.*T* triplex with higher affinity (Kd = 0.75 ± 0.07 nM), in comparison with CTG_hairpin_ (Kd = 1.88 ± 0.13 nM) (Fig 1C). But as expected, CON (Kd = 0.58 ± 0.05 nM) was the best substrate.

### Role of core and C-terminal DNA binding domains for p53 T.A.*T* triplex recognition

**Fig 2:**
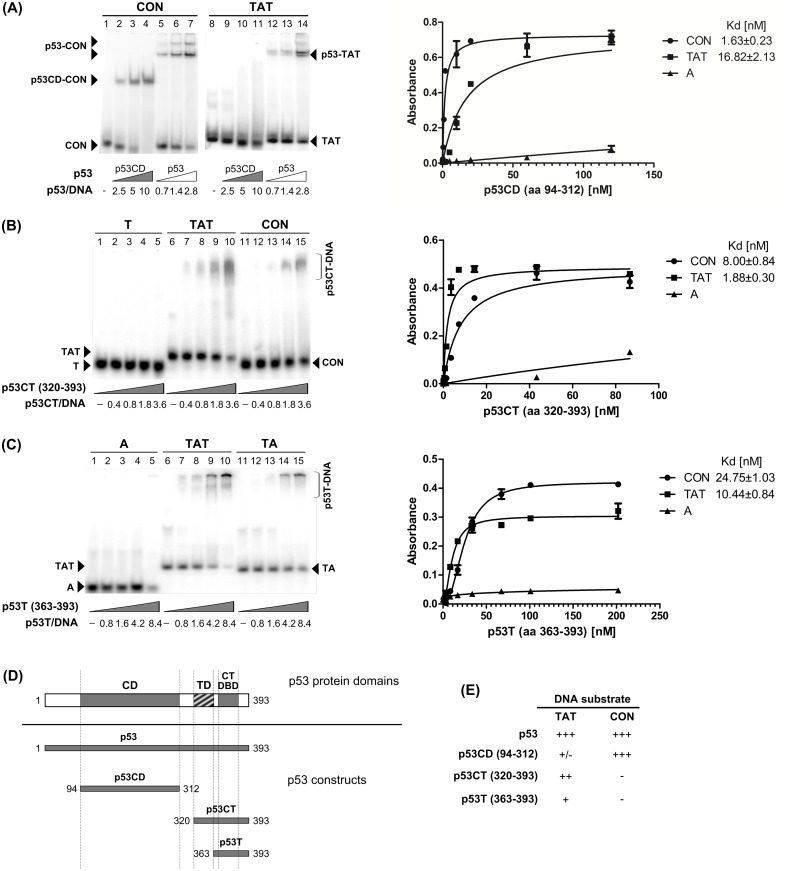
Binding of p53CD and C-terminal p53 fragments to T.A.*T* triplex. **(A)** p53 Core domain (p53CD, aa 94–312) and full length p53 were bound to CON, (lanes 1–7) and triplex (TAT, lanes 8–14) in p53 tetramer/DNA molar ratios 0.7–10 in presence of 10 ng competitor DNA. Graph of p53CD (aa 94–312) binding to biotinylated oligonucleotides by ELISA. p53CD binding curves for the TAT, CON and A oligonucleotides are shown, and the dissociation constants (Kd) are indicated. **(B)** C-terminal part of p53 (p53CT, aa 320–393) was incubated with (dT)_50_ (T, lanes 1–5), triplex (dT)_50_.(dA)_50_.(dT)_50_ (TAT, lanes 6–10) and CON (lanes 11–15) in p53CT tetramer/DNA molar ratios 0.4–3.6. Graph p53CT (aa 320–393) binding to biotinylated oligonucleotides by ELISA. p53CT binding curves for the TAT, CON and A oligonucleotides are shown, and the dissociation constants (Kd) are indicated. **(C)** C-terminal part of p53 (p53T, aa 363–393) was incubated with (dA)_50_ (A, lanes 1–5), triplex (dT)_50_.(dA)_50_.(dT)_50_ (TAT, lanes 6–10) and double-stranded TA (lanes 11–15) in p53CT tetramer/DNA molar ratios 0.8–8.4. Graph of p53T (aa 363–393) binding to biotinylated oligonucleotides by ELISA. p53T binding curves for the TAT, CON and A oligonucleotides are shown, and the dissociation constants (Kd) are indicated **(D)** Scheme showing p53 domains and p53 protein constructs used in this work. **(E)** Relative binding properties of p53 protein constructs to TAT triplex and CON oligonucleotides.

To examine the roles of both DNA binding domains in p53 T.A.*T* triplex recognition we analyzed the interaction of isolated p53 core domain (p53CD, aa 94–312, Fig 2A), C-terminal segment of p53 (p53CT aa 320–393; containing p53CTDBD and tetramerization domains, Fig 2B) and fragment of the last 30 aa of p53 (p53T, aa 363–393, Fig 2C) [10, 29]. At first, we compared binding of p53CD (Fig 2A, lanes 9–11) and full length p53 (Fig 2A, lanes 12–14) to TAT. An unchanged amount of proteins was used for p53CD and p53 binding to CON (Fig 2A, lanes 2–7). In contrast to p53, p53CD was unable to form a stable complex with T.A.*T* triplex.

Binding of C-terminal p53 fragments p53CT (aa 320–393, Fig 2B) and p53T (aa 363–393, Fig 2C) to T.A.*T* triplex was compared with proteins binding to other forms of DNA (ssDNA, dsDNA). We observed that binding of both p53CT and p53T to T.A.*T* triplex DNA was stronger than to the used dsDNA or ssDNA substrates. To better characterize differences in affinities of isolated DNA binding domains to T.A.*T* triplex, we used ELISA with all p53 constructs (p53CD, p53CT and p53T, Fig 2A–2D) followed by quantitation using a specific antibody as was recently described for p53-telomeric quadruplex DNA-binding [13]. With this system, we demonstrated that construct with CTDBD and tetramerisation domain, p53CT (Fig 2B) binds to T.A.*T* triplex with nanomolar affinity (Kd = 1.88 ± 0.30 nM). p53T construct with CTDBD and lacking the tetramerization domain recognized TAT with lower affinity (Kd = 10.44 ± 0.84 nM) than p53CT which is still better than for dsDNA or ssDNA (Fig 2C). And, the lowest affinity for TAT triplex was observed for p53CD (Kd = 16.82 ± 2.13 nM). The results of binding studies are summarized on Fig 2E. Our results showed that the C-terminal DNA binding domain with the tetramerization domain is crucial for TAT triplex high affinity binding.

We confirmed that the C-terminal DNA binding domain is necessary for T.A.*T* triplex recognition by full-length protein with monoclonal antibodies targeting N- and C- terminus (S2A Fig). CTDBD mapping antibody inhibition of p53-non-B DNA complex was previously shown for CTG_hairpins_ and stem-loop structures [33]. DO1, monoclonal antibody targeting aa 20–25 on N-terminus, supershifted both p53-CON and p53-TAT complexes (S2B Fig, lanes 3,8). In contrast to DO1, PAb421 antibody (mapping CTDBD, aa 371–380) induced a partial inhibition of p53 binding to TAT triplex (S2B Fig, lane 9) as opposed to supershifting of p53-CON (S2B Fig, lane 4). ICA9, mapping aa 388–393 on extreme C-terminus, supershifted both p53-CON and p53-TAT complexes (S2B Fig).

### Binding of p53 to triplex forming sequence in supercoiled DNA *in vitro*

**Fig 3:**
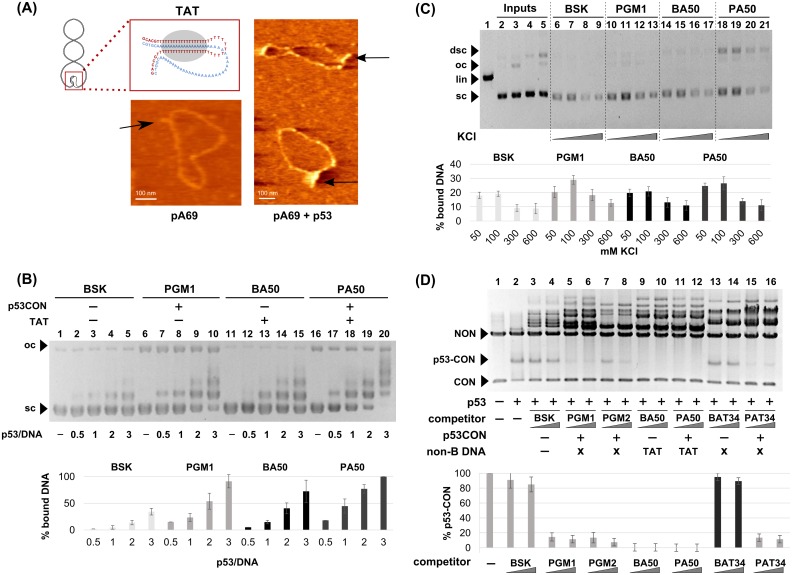
Binding of p53 to supercoiled DNA bearing homoadenine-homothymine triplex forming sequences. **(A)** Scheme of intramolecular T.A.*T* triplex in scDNA. AFM image of sc pA69 plasmid adsorbed on mica surface in the presence of 2 mM MgCl_2_ and complex of pA69 with p53. **(B)** Comparison of p53 binding to scDNA with and without triplex forming sequence (dA)_50_.(dT)_50_ by EMSA. Binding of p53 protein to pBSK, pPGM1, pBA50 and pPA50 detected by EMSA in agarose gel. p53 protein was bound to scDNA (pBSK, 200 ng, lanes 1–5), scDNA with CON (scPGM1, 200 ng, 6–10), scDNA with (dA)_50_.(dT)_50_ (scBA50, 200 ng, 11–15) and scDNA with both CON and (dA)_50_.(dT)_50_ (scPA50, 200 ng, 16–20) in p53/DNA molar ratios 1–3 at 4°C, EMSA was performed at 4°C. Graph represents the dependence of percents of bound DNA on the amount of p53 proteins calculated from three experiments. **(C)** Interaction of p53 with scDNA (BSK, PGM1, BA50 and PA50) in presence of pBSK/SmaI (linear competitor, lin) by immunoprecipitation on MBG. Agarose gel electrophoresis of DNA recovered from MBG after incubation of DO1-wtp53-DNA complex at the beads to 50, 100, 300 or 600 mM KCl for 30 min at 10°C followed by the SDS treatment. DNA inputs of scDNA BSK (lane 2), PGM1 (lane 3), BA50 (lane 4), PA50 (lane 5), linBSK (lane 1). Arrows indicate precipitated supercoiled (sc), open circular (oc), linear (lin) and supercoiled dimers (dimer sc). Mean values of bound DNA from three independent experiments were plotted in the graph. Graph represents the dependence of percents of bound DNA on the concentration of KCl in washing buffer calculated from three experiments. **(D)** Competition assay of p53 binding to CON and non-B-DNA structures in scDNA plasmids. First, full length p53 (60 ng) was incubated with 200 ng PGM1/*Pvu*II fragments (short fragment with CON sequence (CON, 474 bp) and long fragment as linear nonspecific competitor (NON, 2513 bp) for 20 min on ice to form p53-CON complexes. Subsequently, 200 or 300 ng of different scDNA plasmid competitors were added and incubation was prolonged to 40 min. Plasmids forming triplex T.A.*T* were marked by TAT, plasmids forming cruciform by X. Graph represents the dependence of percents of bound DNA on the amount of used competitor scDNAs calculated from three experiments.

Intramolecular T.A.*T* triplex (H-DNA) formation in the presence of Mg^2+^ ions in supercoiled plasmids containing homoadenine-homothymine blocks has been described for several vectors [35, 47]. We prepared constructs based on the pBSK vector in variants with and without p53 specific sequence (CON), triplex-forming sequence (TFS, (dA)_50_.(dT)_50_) and AT-rich cruciform-forming sequence d(AT)_34_ (more details in S1 Table). Formation of non-B DNA structures in different superhelical plasmids was checked by several techniques (S3 Fig): S1 nuclease treatment, OsO_4_-bipy modification detected by specific antibody against OsO_4_-bipy-DNA adducts [48] (S3F Fig) and OsO_4_-bipy modification on the sequencing level [47, 49] (S3G Fig). The H-DNAs formed in plasmids pBA50 and pA69 were also visualized by AFM (Fig 3A and S5 Fig).

At first, we compared p53 binding to scDNA capable of H-DNA formation at native superhelical density pBA50 and pPA50 with other plasmids pBSK and pPGM1 by EMSA (Fig 3B). Differences in p53 recognition of scDNA with and without TFS or CON are measurable by number and intensity of retarded bands (compare lanes 3, 8, 13 and 18, Fig 3B) and were evaluated by densitometry of the band corresponding to free (protein-unbound) DNA. The fraction of DNA bound by the protein was calculated and plotted in the graphs shown in Fig 3B (average of at least 3 independent experiments). Both plasmids pPGM1 (with CON, lanes 7–10) and pBA50 (with TFS and H-DNA potential, lanes 12–15) were more strongly bound by p53 than pBSK (Fig 3B, lanes 2–5), similarly to pA69 (with H-DNA potential) versus pUC19 (S4 Fig). The best substrate for p53 was pPA50, plasmid with both motifs CON and TFS (Fig 3B, lanes 16–20).

Furthermore, we applied a competition immunoprecipitation assay and compared binding of p53 to scDNA with and without TFS and CON in the presence of competitor DNA (pBSK/*Sma*I). Increasing salt concentration (50–600 mM KCl, [50]) was applied to detect the difference in stabilities of p53-scDNA complexes containing CON and TFS (Fig 3C). We observed an increase in stability of p53-scDNA binding in the presence of TFS and in agreement with other results, more so in the case of CON (Fig 3B). Due to stability of p53-scDNA complex we were able to perform AFM visualization of p53 bound to scDNA with triplex-forming sequence (dA)_69_.(dT)_69_ is depicted in Fig 3A and S5 Fig.

To probe differences in relative p53 binding affinity to scDNA with/without TFS and CON we used a competition assay proposed previously [30]. Binding of the p53 protein to CON fragment yielded a well resolved retarded band p53-CON (Fig 3D, lane 2). The intensity of this band was affected by the additions of tested scDNAs, which represented the competitors. Decrease of the p53-CON band intensity relative to the intensity detected in the absence of the competitors reflected the relative affinity of p53 for a given competitor, bar graph represents results from three independent experiments. We observed that pBA50 (T.A.*T*, H-DNA) was a comparable competitor to all plasmids with CON (pPGM1, pPA50 and pPAT34). The control vector pBSK together with pBAT34 (X, cruciform DNA) were the worst competitors.

### *In-silico* screening of human regulatory sequences for co-occurrence of CON binding sites and potential T.A.T triplex-forming sequences

**Fig 4:**
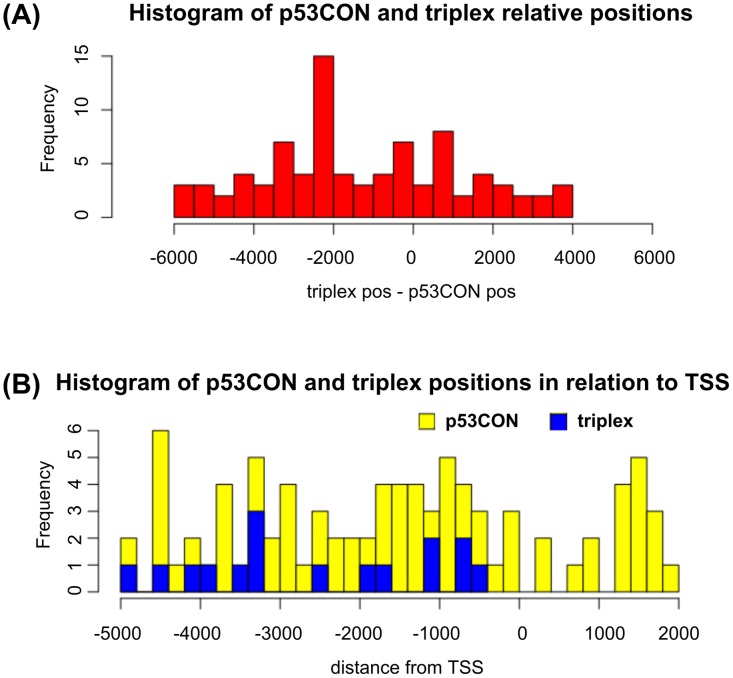
T.A.T triplex and p53CON positions in promoters of the 43 analyzed human genes. **(A)** Relative distance between each p53CON and the corresponding T.A.*T* triplex. Most p53CONs are 2000-2500bp downstream of the triplex. Second peak corresponds to T.A.*T* triplex positioned in front CON. **(B)** Absolute positions of p53CONs (yellow) and T.A.*T* triplex-forming sequences (blue). TSS is positioned at 0.

To investigate the possible significance of p53 binding of T.A.*T* triplex-forming sequences for transcription regulation we carried out a series of *in-silico* investigations. Within the context of p53 transcription factor functions involving CON recognition, we looked for T.A.*T* triplex-forming and CON sequence co-occurrence in the human genome to predict new class of p53 target genes. We analyzed the -5000/+2000 bp neighborhoods of 42106 RefSeq gene transcripts (promoters). Of these, 19373 promoters were found to contain at least one CON sequence when 1 mismatch was allowed. T.A.*T* triplex-forming sequences with a prevailing poly(A) or poly(T) run with score> = 18 were found in 376 sequences. Because of the asymmetry in occurrence of these two patterns we decided to screen the promoters primarily on the predicted length of the T.A.*T* triplex. There were 43 promoters of candidate p53 target genes with at least one CON and a T.A.*T* triplex with a poly(A/T) run longer than 40 bp. S2 Table shows locations, common gene abbreviations and binding site data for these promoters. Interestingly, *in-silico* analysis shows that most CONs are downstream of the triplex (Fig 4).

STRING-db functional association tool shows 16 of the 43 highest-scoring genes/proteins found in the screening, together with p53 and 10 most-related proteins from STRING-db, organized into a network by common properties and interactions (S6 Fig). 16 proteins from our study that are also part of well-connected networks are: ABCG5, PIK3R4, INSR, MIB1, MAPK9, TGIF1, STAG2, NFAT5, MAK16, DDX54, NAT10, BMS1, PSMB2, PEX12, MCC and MCCC1 shown in blue (S6 Fig). The common functional theme for the proteins clustered by STRING as suggested by gProfiler GO term enrichment analysis is “regulation of signal transduction” (P-value = 2.52e-04).

### Triplex forming sequence and DNA topology influence p53 transactivation

**Fig 5:**
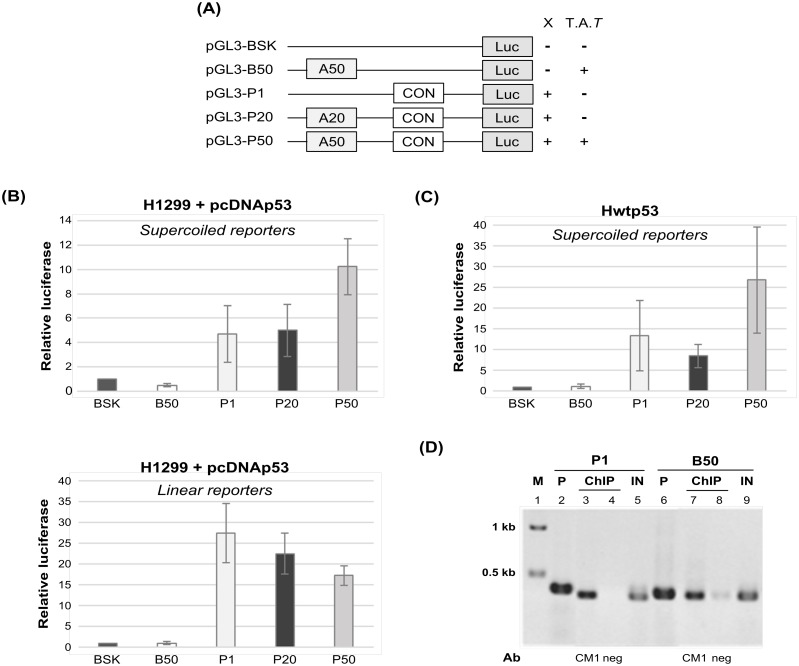
Influence of T.A.*T* triplex forming sequence on p53-driven activation of CON containing reporter vector in scDNA and lin DNA. **(A)** Scheme of reporter plasmid constructs used in luciferase reporter assay and non-B DNAs formation under supercoiled stress (CF- cruciform, TAT-triplex). **(B-C)** H1299 cells were transiently transfected with plasmids expressing the p53 (pCDNA3.1-p53) or pCDNA3.1 vector alone (CMV) together with reporter: the supercoiled or linear reporter plasmids (BSK, P1, P20, P50, B50) expressing the firefly luciferase gene and a reference plasmid with the renilla gene under control of the SV40 promoter. Luciferase activity was analyzed 16 hours after transfection and signal was normalized on renilla signal. Transfections were carried out in triplicates at least at three independent times and standard deviations are indicated. **(B)** p53 activation of supercoiled reporters. Luciferase activity was normalized on control with vector alone. Only B50 and P50 reporters were able to form triplexes. p53 activation of linear reporter as described above, none of used reporters was able to form triplexes. **(C)** p53 activation of supercoiled reporter plasmids in H1299-wtp53 cells (Tet-on promoter). Luciferase signal after p53 induction was normalized on control without p53 induction. Only B50 and P50 reporters were able to form triplexes. **(D)** Interaction of full length p53 with CON (P1) and triplex T.A.*T* (B50) in scDNA plasmids by ChIP *in vivo*. Plasmids BA50 or PGM1 (2 μg) were transfected into H1299 cells together with vector pCDNA3.1-wtp53 (0.1 μg). ChIP was performed with CM1 antibody. Results of PCR analyses of immunoprecipitated DNA were detected on a 1.5% agarose gel in 1× TAE buffer. PCR samples on the gel are: marker (lane 1), plasmid PGM1 (P1, lane 2) and BA50 (lane 6); 1/20 of DNA input (lanes 5 and 9 marked as IN); IP with IgG (negative control) (lanes 4 and 8); IP with CM1 Ab (lanes 3 and 7).

To analyze whether the triplex-forming sequence (dA)_50_ has any effect on p53-driven transcription we performed luciferase reporter assays using reporter vectors in variants with and without TFS (dA)_50_, (dA)_20_ too short for triplex formation and p53 specific sequence CON (Fig 5A). Luciferase assay was performed in H1299 cells with transfected pCDNAp53 effector and related to transfected pCDNA vector only (Fig 5B) with linear and supercoiled reporter vectors and in p53 inducible H1299wtp53 cell line (Tet-on system) with sc reporters after p53 induction and related to no induced stage (Fig 5C). Only supercoiled reporters could form non-B DNA structures, in our case H-DNA (Fig 5A, 5B and 5C; B50, P50, TAT) or cruciform (Fig 5A, 5B and 5C; P1, P20, cruciform-X). As expected p53 expression resulted in stronger activation of all vectors containing CON (P1, P20, P50) in comparison with vectors missing CON (BSK and B50). As for P20, with an insert not yet suitable for triplex formation [35], the activation was comparable to the original reporter P1. Interestingly, activation of P50, for intramolecular triplex formation already satisfactory reporter occurring when the reporter was supercoiled, was significantly stronger than analogous reporter containing only CON (P1) (Fig 5B and 5C). For linear reporter P50 such effect was not observed (Fig 5B). In the case of B50, a repression was observed with sc form of reporter (Fig 5B and 5C). In summary, triplex-forming sequence (dA)_50_ enhances p53-driven transcription from supercoiled reporter containing p53 specific sequence CON.

To confirm *in vivo* p53 binding to (dA)_50_ sequence capable to form H-DNA, supercoiled plasmids B50 (H-DNA potential) and P1 (CON with potential to form DNA cruciform) were transfected to H1299 cells together with effector plasmid pCDNA3.1p53 and a ChIP assay was performed with p53 specific antibody CM1 (Fig 5D lane 3 and 7) and IgG (negative control) (Fig 5D, lane 4 and 8). We observed comparable binding of p53 to B50 (TAT, H-DNA-forming sequence Fig 5D, lane 7) as to P1 (CON, Fig 5D, lane 3).

Together, these data demonstrate that the triplex-forming sequence (dA)_50_ under conditions favorable for the actual H-DNA formation can influence the level of DNA-binding and transactivation of p53 binding sites in promoter regions by p53 *in vivo*.

### Analysis of candidate p53 target genes with triplex-forming sequences in promoter region

To better prioritize the candidate p53 target genes identified by the above *in-silico* screening (S2 Table and S6 Fig) we consulted publicly available microarray and sequencing datasets for experiments involving full-length p53, p53CΔ30 and p53S389A transformed cells originally lacking p53 [51, 52] or experiments with endogenous p53 activated by nutlin-3/doxorubicin/5-fluoruracil for gene expression values [42, 53–58], results are summarised in S3 Table. This way we were able to evaluate expression of many of the candidate p53 target genes and also evaluate the influence of p53 C-terminus as shown in S3 Table. Several of the genes selected by the screen showed consistent up-regulation in these conditions (*MCC*, *NFAT5*, *ENOX1*, *ABCG5*) or down-regulation (*MAPK9*, *MAK16*). Interestingly, *NAT10* and *STAG2* belongs to several genes down-regulated after activation of p53 by drug treatment and up-regulated in p53 overexpression in p53 null cells. Several up or down regulated genes (*ABCG5*, *INSR*, *MCC*, *NFAT5* and *NAT10*) were limited to the STRING-db-supported functionally associated group of genes. Intact C-terminus was necessary for strong p53-dependent activation of *MCC*, one of the best candidate p53 target gene, in contrast to well-known target gene *MDM2* (S3 Table).

**Fig 6:**
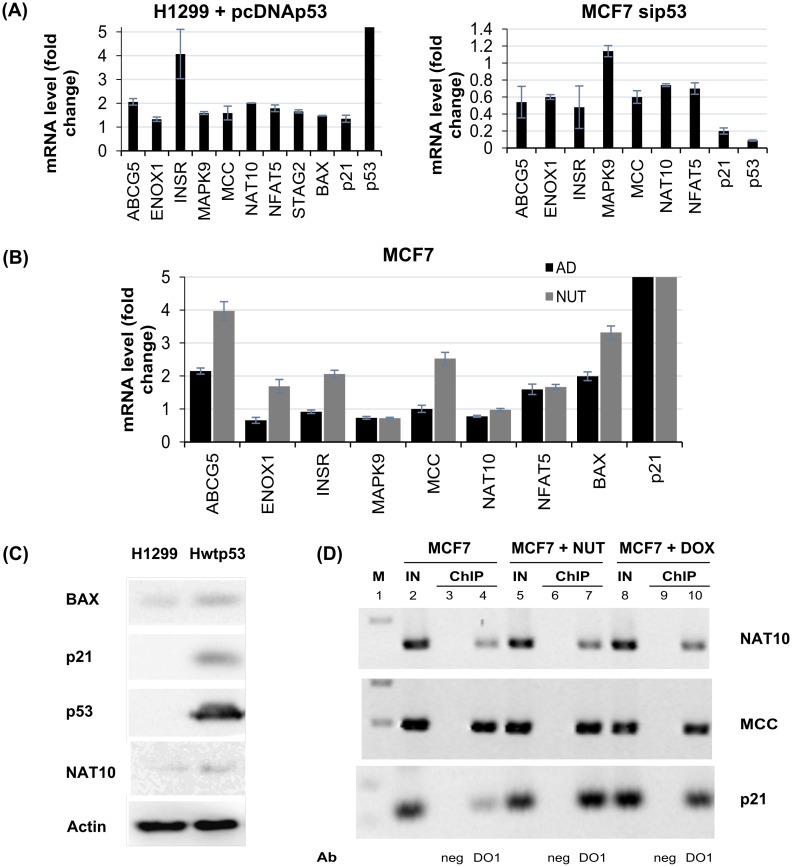
Verification of candidate p53 target genes. **(A)** RT-qPCR analysis of candidate p53 target genes and BAX, p21 and p53 mRNA levels in i) H1299 cells transfected by pCDNAp53 for 48 hours (left graph); ii) MCF7 cells with downregulation of p53 by siRNA over control siRNA for 48 hours (right graph). **(B)** RT-qPCR analysis of candidate p53 target genes and *BAX*, *p21* mRNA levels in MCF7 cells after nutlin-3 or actinomycin D 12 hours treatment. Gene values were normalized to GAPDH. The values are the average of three independent experiments. **(C)** p53 mediated up-regulation of NAT10 on protein level and activation of BAX and CDKN1A was analyzed in Hwtp53 cells (24 hours induction) vs H1299 without p53 expression. Western blots presenting protein levels of p53, NAT10, CDKN1A and BAX. Actin was used as loading control. **(D)** Chromatin immunoprecipitation showing p53 binding to *MCC* and *NAT10* promoters which contain a TAT triplex motif. DNA fragments from MCF7 cells without and with nutlin-3/doxorubicin 4 hours treatment were immunoprecipitated using DO1 antibody against p53 (lane 4,7,10), negative control ChIP with IgG (lanes 3,6,9), positive input control (1/15 input for ChIP, lanes 2,5 and 8).

To validate experimentally our set of candidate genes (S2 Table) as novel p53 target genes, at first we performed their RT-qPCR analysis after p53 transient transfection experiment in p53 null cell line (H1299, Fig 6A, S3 Table). As expected p53 overexpression activated *p21*, *BAX* and several new potential candidate p53 target genes (e.g. *ABCG5*, *INSR*, *MCC*, *NFAT5*; Fig 6A). Next, we checked whether p53 downregulation in MCF7 cells could reduce their expression. Downregulation after p53siRNA treatment was observed for *ABCG5*, *ENOX1*, *INSR*, *MCC*, *NAT10* and *NFAT5* (Fig 6A, S3 Table). In addition, *ABCG5*, *ENOX1*, *INSR*, *MCC*, *NFAT5* together with *p21* and *BAX* were induced in MCF7 cells treated with nutlin-3, a p53-stabilizing agent (Fig 6B). However, activating p53 by actinomycin D did not promote *ENOX1*, *INSR*, *MCC* expression, in contrast to *BAX*, *p21* and *ABCG5* (Fig 6B). For another candidate genes *MAPK9* and *NAT10* we observed down-regulation after p53 activation by actinomycin D drug treatment. Interestingly, after 24 hours tetracycline p53 induction of Hwtp53 cells, we observed activation of NAT10, p21 and BAX on the protein level (Fig 6C). To determine binding of endogenous p53 to triplex forming sequences in selected new potential p53 target gene promoters, we performed ChIP assay for analysis of p53 binding on *MCC*, *NAT10* and *p21* promoters in MCF7 cells (Fig 6D). Using of primers covering TAT triplex we observed p53 binding to *MCC* and *NAT10* promoters also after stabilization of p53 after nutlin-3 and doxorubicin treatment in MCF7 cells (Fig 6D). Taken together, *in silico* analysis of expression data, RT-qPCR and ChIP analysis have shown connection between p53 and new set of potential p53 target genes with triplex forming sequences in promoter regions.

## Discussion

Alternative, non-B DNA structures, such as triplex, quadruplex, hairpin and cruciform can be formed by sequences that are widely distributed throughout the human genome [59]. Triplexes and cruciforms are implicated in regulating gene expression and causing genomic instability [60, 61]. Despite the known fact of tumor suppressor p53 protein importance for maintaining genomic stability, the mechanisms in this protective function are still not well understood.

Regions with the potential to form triplex DNA are generally over-represented in the promoter regions and introns of genes involved in cell signaling as indicated by genome-wide bioinformatics analyses [18, 19, 62]. In our previous bioinformatics study, we showed the prevalence of the T.A.*T* triplex class in the human genome [19]. The present work was a follow-up by focusing on p53 recognition of T.A.*T* triplex-forming sequence (dA)_50_.(dT)_50_, especially in promoters containing this sequence in close proximity to specific p53 binding sites (CONs).

A number of independent studies have established that p53 recognizes non-B DNA structures including hairpins, stem-loops, cruciforms, mismatches, bulges, G-quadruplexes, three- and four-way junctions [4, 30, 31, 63–66]. For example CTG.CAG trinucleotide repeats were shown to be a novel class of p53-binding sites *in vitro* and *in vivo*, CTG and CAG hairpins were determined as p53 bound non-B DNA structures in that repetitive sequence [33]. To best of our knowledge no study has been published on triplex DNA recognition by wild-type p53 protein. Mutant p53 (R273H) binding to genomic fragment containing mirror repeats with the potential to form intramolecular triplex was shown in an earlier study of ours on identifying natural binding sites in glioblastoma cell line U251 [67].

In the present study, a range of biophysical approaches was used to analyze the interaction of full-length and isolated DNA binding domains of p53 with intermolecular triplex DNA. The T.A.*T* type of triplex was chosen with respect to physiological conditions necessary for triplex formation [35, 47] and for the high frequency of potential triplex-forming sequences in the genome [39]. Both EMSA and ELISA assays demonstrate slightly greater binding affinity of full-length p53 protein to the T.A.*T* triplex than to the CTG_hairpin_ (Fig 1). Binding of full-length p53 to T.A.*T* triplex was weaker than to specific sequence CON. In contrast to p53T and p53CD, the affinity of p53CT for the T.A.*T* triplex was in range of full-length p53. Thus, our data showed that both CTDBD and the tetramerization domain (aa 325–356) are necessary for high affinity p53 binding to the T.A.*T* triplex.

Although binding of DNA by the C-terminus is usually marked as non-specific, CTDBD has a major role in non-B DNA structures recognition (e.g. stem-loop structure, G-quadruplex, CTG and CAG hairpins, [13, 31, 33, 68]) and there is increasing evidence for the importance of intact CTDBD for regulating sequence-specific DNA binding, transactivation and also for the maintaining genomic stability [69, 70]. The C-terminus is marked by the presence of a large number of positively charged amino acid residues and has an inherently disordered character. The CTDBD structure gives intrinsic flexibility and possesses molecular recognition features necessary for the multifunctional nature of this region [70, 71]. The formation of a partially helical structure was observed experimentally after binding of the C-terminus to non-specific DNA (sheared herring sperm DNA, [72]). Laptenko´s recent *in vivo* and *in vitro* study with p53 proteins mutated in CTDBD (mimicking acetylation/phosphorylation) points to several positive roles of intact unmodified CTDBD in regulating sequence specific DNA binding, p53 protein stability, p53 cellular localization and co-factor recruitment [70]. Recently, the relevance of post-translational modifications of the C-terminus in the DNA-binding properties of p53 has been reviewed in [71].

There is no systematic study to date of the role of DNA binding domains in different non-B DNA structures recognition. CTDBD is necessary for recognition of DNA cruciform and stem-loop structures both formed by CON sequences [30, 31], as well as CTG.CAG tracts [33]. In the case of p53 interaction with scDNA, we have shown that at least the dimeric form of CTDBD is essential for highly selective binding [10]. Three-stranded junctions (with and without mismatches) were recognized by full length protein but with lower affinity by p53CΔ30 (containing core domain with the tetramerization domain) as well [4]. On the other hand, the CD and dimerization domain are required for high affinity interaction with insertion/deletion lesions [5]. Our data agree with the majority of studies on p53 interaction with alternative DNA structures, showing the CTDBD and tetramerization domain is responsible for high-selective binding of p53 to non-B DNA structures [4, 9, 12, 30–32, 73].

For the first time we show preferential p53 binding to supercoiled plasmids capable of H-DNA formation by (dA)_50_.(dT)_50_ sequence. We verified H-DNA formation under superhelical stress under conditions used for p53 binding using several techniques and visualized them by AFM. scDNA pBA50 was somewhat more weakly bound by p53 than scDNA with CON (pPGM1). In competition assay, pBA50 and pPA50 capable of H-DNA formation were better competitors than pBAT_34_ forming AT-rich cruciform and comparable in competition to plasmids with CON (Fig 3D). Supercoiled pPGM1 was shown to form cruciform by CON with stem-loop motif with mismatches and to be more attractive for p53 binding [30, 31]. We suspect that the high affinity of p53 for scDNA capable of forming H-DNA is due to the fact that besides the triple-helical part of the scDNA molecule (Fig 3A), p53 also recognizes single-stranded loops and junctions (Fig 3A) already described as p53 recognition motifs in DNA [31].

Identification of T.A.*T* triplex as a novel p53 binding site recognized by CTDBD raises the question of the physiological significance of such interaction. The nM binding/dissociation constant that we observed for p53 binding to intermolecular T.A.*T* triplex (Fig 1C) shows that this binding is slightly stronger than to CTG_hairpin_ and slightly weaker than to CON observed in this work using ELISA and EMSA (Fig 1) providing evidence for the *in vivo* relevance T.A.*T* triplex p53 binding. The nM range of binding/dissociation constant for p53 sequence-specific interaction has been found by several groups using various techniques e.g. Fersht´s group by FA [30, 31]. For sequence-specific p53 binding, application of competitive fluoresce anisotropy technique has shown Kd values in the range of 10–100 nM. The pM dissociation constant for sequence-specific and insertion/deletion lesion p53 interactions has been reported so far in only one study [5].

We speculate that the T.A.*T* triplex formed by (dA)_50_.(dT)_50_ tracts may act as a non-B DNA p53 binding site essential for p53 stability, co-factor recruitment and regulating sequence-specific binding mainly in the case of unmodified C-terminus by phosphorylation and acetylation. Binding of p53 to a significant number of sites within the genome depends on the availability of unmodified CTDBD according to a recent report [70]. The C-terminus has been shown to be crucial for the sliding mechanism of p53 recognition of CON by p53CD [74]. p53 binding to multiple non-B binding sites can influence their stability. One suggested scenario is that non-B DNA structures may be targeted by p53, which then binds to and stabilizes or destabilizes such DNA structures to increase gene transcription. Besides its effect on gene transcription, p53-non-B DNA recognition can participate in DNA repair, DNA replication and/or DNA recombination. Genome-wide studies show that p53 binds to many loci in the genome, including sites not associated with transcriptional control [75]. Recently, the prevention of accumulation of DNA damage by p53 binding to subtelomeric regions has been described [76]. Walter et al. showed that p53 induces local distortions in mismatched trinucleotide repeats and suggested that p53 may be involved in the maintenance of CTG.CAG tract stability [12, 30, 33]. In our case we observed a positive effect of T.A.*T* triplex-forming sequence (dA)_50_.(dT)_50_ on the stability of the p53-scDNA complex and p53 binding to (dA)_50_.(dT)_50_ in scDNA in cells. For this reason, we hypothesize that p53 interaction with T.A.*T* triplex, primarily by CTDBD, can stabilize p53 protein in both non-B DNA and CON. Additionally, we can discuss the role of the p53-T.A.*T* triplex recognition in the process of DNA repair. It was shown that triplex-forming oligonucleotides are able to activate DNA recombination and DNA repair in addition to inducing genomic instability [77]. Intact p53 C-terminus is necessary for recognition of damaged DNA and recombination intermediates [2, 3, 7, 8, 63, 78, 79]. Triplex DNA may also elicit genetic instability by a roadblock to DNA replication and transcription elongation [80]. The DNA damage tolerance pathway and p53 regulates DNA replication fork progression according to a recent study [78]. It was shown, that the helical distortions and structural alternations induced by triplex formation may be recognized as “DNA damage” [80, 81]. So far, we can only speculate that p53-T.A.*T* triplex recognition can eliminate DNA damage caused by triplex formation.

Interestingly, the group of proteins specifically recognizing triplex DNA (HMG, helicases, RAD51, RPA [82]) are also known as p53 interaction partners. As large number of p53 interacting proteins also interact with triplex DNA, we reason that p53 triplex recognition has the potential to influence the regulation of genomic stability, DNA repair, DNA replication, DNA recombination and gene expression at different levels.

Using luciferase reporter assay in two different cell systems, we demonstrate that T.A.*T* triplex-forming sequences (dA)_50_.(dT)_50_ in front of CON, enhanced promoter activation by p53. Interestingly, the reporter vector containing only T.A.*T* triplex-forming sequence (dA)_50_.(dT)_50_ was repressed by p53 protein. Both these effects suggested that T.A.*T* triplex-forming sequences have the potential to influence transcription in both directions. We assume that positioning of p53 on promoter region facilitates p53 recognition and transcription of genes.

Our *in-silico* analysis with STRING showed that a fraction of promoters containing both CON and a potential T.A.*T* triplex-forming sequence belong to the functional and structural association network of p53. Although p53 has a large association network, repeated experiments with randomly chosen UniProt Ids have shown that the majority of blind tests had networks with less than 10 interactions while we observed 14, before adding the additional 10 best connected proteins. A medium strength enrichment (P-value ~ 0.00025 after correction for multiple testing) was obtained from gProfiler for the most enriched Gene Ontology term: “regulation of signal transduction”. Consequently, the *in-silico* experiments did not yield results that would have the power of proof for us. Rather, they should be viewed as a tool to narrow down possible candidates for further studies, such as the RT-qPCR experiments carried out here. Several candidate genes from the narrowed-down list that have been tested by RT-qPCR show increased expression in p53 dependent manner in p53 null cell line. The best candidates are *ABCG5*, *ENOX1*, *INSR*, *MCC*, *NAT10*, *NFAT5* and *MAPK9* (Fig 6). Only *MCC*, *INSR* and *NAT10* association with p53 has been described so far. *MCC* was described as a target gene upregulated by nutlin-3 but not by doxorubicin and its promoter CON sequence was bound by p53 in U2OS cells [83]. *INSR* is described as a target gene upregulated by overexpression of p53 in HCT116 p53-/- cells [83]. Recently, NAT10 was described as a protein regulating p53 activation through its acetylation and also that NAT10 was upregulated under stress conditions in a p53-dependent manner. Thus, NAT10 forms a positive regulation feedback with p53 in response to stress [84].

The tumor suppressor p53 has been studied extensively as a direct transcription regulator of several hundred target genes and it is currently known to indirectly regulate thousands of genes [85]. Detailed promoter analyses of each potential candidate p53 target gene have to be done to validate them as genuine p53 target genes, as well as, to prove the importance of DNA triplex formation for their regulation by p53. So far, *in-silico* analysis of promoters of candidate p53 target genes shows that most CONs are downstream of the triplex and we can only speculate about the possible functions of T.A.*T* triplex-forming sequence as enhancers and this has to be experimentally proven. Recently, p53 recognition of regulatory enhancer elements within the non-coding genome was identified in human fibroblasts [86]. p53 has been shown to regulate the expression of multiple genes over long distances via looping and binding to enhancers [85]. Originally, we showed that p53 is involved in DNA looping *in vitro* [87]. More experiments with positioning of TAT and CON sequences have to be conducted to confirm this hypothesis.

Genome organization and local DNA structural effects on gene expression are still not sufficiently investigated. Our results show possible concomitant binding modes of p53, where one of them depends on structures that may only be present transiently in the genome. Further studies would provide us with better understanding of the local environment at promoters and new modes of transcriptional regulation.

### Conclusions

In summary, we show that p53 protein possessing intact C-terminus exhibits the ability of p53 to bind with high affinity to intermolecular and intramolecular T.A.*T* triplex DNA. Moreover, T.A.*T* triplex influences transcription from a CON containing reporter and p53 T.A.*T* binding was also detected *in vivo* by chromatin immunoprecipitation techniques. *ABCG5*, *ENOX1*, *INSR*, *MAPK9*, *MCC*, *NAT10* and *NFAT5* were associated with p53, as potential novel p53 target genes with T.A.*T* motif in their promoter.

## Supporting Information

S1 Fig Protein analysis and comparison of binding of full length p53 to T.A.*T* triplex and CON. (A) SDS-PAGE analysis of p53 proteins used in the study. The purity and appropriate size of each proteins was analyzed by Coomassie blue staining of 12.5% SDS-PAGE gel. **(B)** Full length p53 was bound to 1 pmol of ^32^P-labeled 50-mer oligonucleotides represented by p53 nonspecific dsDNA (NON, lanes 1–5), p53 specific dsDNA with CON (CON, lanes 6–10) and triplex (dT)_50_.(dA)_50_.(dT)_50_ (TAT, lanes 11–17) in the presence of DNA competitor (linear plasmid pBSK/*Sma*I, 50 ng). The reactions were separated on 4% 0.5× TBM (2 mM MgCl_2_) polyacrylamide gel (PAGE), 3h. Radioactively labeled DNA was detected by autoradiography. B,C) Full length p53 was bound to 1 pmol of ^32^P-labeled 50-mer oligonucleotides represented by p53 specific dsDNA with CON (CON, B) and triplex (dT)_50_.(dA)_50_.(dT)_50_ (TAT, C) in the presence of DNA competitor (linear plasmid pBSK/*Sma*I, 20 ng). The reactions were separated on 5% 0.5× TBM (2 mM MgCl_2_) PAGE, 1 h. Radiolabeled DNA was detected by autoradiography.
(TIFF)

S2 Fig Interaction of CTDBD with T.A.*T* triplex. The effect of C-terminal modifications of p53 protein by Ab on T.A.*T* triplex recognition. **(A)** Scheme of p53 used in this study, shown as boxes below the map of p53 domains. The evolutionarily conserved domains are indicated: core DNA binding domain (CD; aa ~100–300), tetramerization domain (TD; aa 325–356) and basic C-terminal DNA binding domain (CTDBD; aa 363–382) and location of p53 antibodies PA421, ICA9 and DO1 used in our study. **(B)** Effect of C-terminal modifications of p53 protein by Ab on T.A.*T* recognition. The antibodies (DO1, PAb421 and ICA9; 1.5 μg) were bound to p53 (300 ng) in Ab/p53 molar ratio 2/1 at RT for 15 min. Then 1 pmol of ^32^P-labeled 50-mer oligonucleotides represented by p53 specific dsDNA with p53CON (CON, lanes 1–5) and triplex (dT)_50_.(dA)_50_.(dT)_50_ (TAT, lanes 6–10) were added and mixtures were incubated at 4°C for 20 min. The reactions were separated on 4% 0.5× TBM (2 mM MgCl_2_) PAGE at 4°C. Radioactively labeled DNA was detected by autoradiography. Mouse monoclonal anti-p53 antibodies (mAb) (DO1 (aa 20–25), Bp53 10.1 (aa 375–379), PAb421 (aa 371–380) and ICA9 (aa 388–393)) and anti-GST Ab (G1160, Sigma) were used.
(TIFF)

S3 Fig Non-B DNA structures analysis supercoiled plasmid DNA (pBSK, pPGM1, pPGM2, pBA50, pPA50, pBAT34, pA69 and pPAT34) by S1 treatment, OsO4-bipy modification and its combination with S1 treatment. (A,B,D,E) Scheme of non-B DNA structures detection by S1 nuclease treatment described in [30]. scDNAs were treated with S1 nuclease followed by *Sca*I digestion. Detection of two fragments indicates one major non-B DNA structure (cruciform or triplex) formation in the polycloning site in the case of pBA50 (A), pPGM2 (D, lane 12), pBAT34 (E, lane 4), and pAT34 (E, lane 8). But also pPGM1 (D, lane 8), pBA50 (B; E, lane 12), pPA50 (E, lane 16) and pBSK (D, lane 4) were sensitive to S1 nuclease treatment; two pairs of fragments (black lines) were detected, indicating that all plasmids can form non-B DNA structures with unpaired bases. (C) AFM visualization of intramolecular triplex in pBA50, conditions as described in Fig 2. (F) Detection of non-B DNA modified with OsO_4_-bipy by dot blot on nitrocellulose membrane with specific antibody against OsO_4_-bipy-DNA adduct as described in [48]. pUC19 (vector only) and pA69 were modified by condition described in [48]; (G) Detection of non-B DNA in plasmid DNA pre-incubated in 20 mM TrisHCl pH8, 2mM MgCl_2_ without/with 100 mM NaCl by OsO_4_-bipy modification followed by primer extension analysis of pBSK (1,2), PGM1 (3,4), PGM2 (9,10), pBA50 (11,12) plasmid DNA, conditions described in [47]. Primer extension from T7 primer was used. See S1 File for experimental details.
(TIFF)

S4 Fig Comparison of p53 binding to scDNA with and without triplex forming sequence (dA)_69_.(dT)_69_ by EMSA. Binding of p53 protein to pUC19 and pA69 detected by EMSA in agarose gel. p53 protein was bound to scDNA (pUC19, 200 ng, lanes 1–5) and scDNA with (dA)_69_(dT)_69_ (pA69, 200 ng, 6–10) in p53/DNA molar ratios 1–5 at 25°C, EMSA was performed at 4°C.
(TIFF)

S5 Fig AFM visualization of plasmids containing triplex-forming sequences and their complexes with p53 proteins. **(A)** AFM image of scBA50 plasmid mounted in the presence of 5 mM MgCl_2_. Scale bar represents 200 nm. **(B)** Image of pA69 complexes with p53, proteins were incubated with DNA in molar ratio 5/1 in DNA binding buffer and then loaded on mica surface in the presence of 5 mM MgCl_2_. Scale bar represents 500 nm. **(C)** pA69 plasmid with p53 proteins in 3D projection.
(TIFF)

S6 Fig STRING-db analysis of the highest-scoring proteins of candidate p53 target genes. The 43 highest-scoring proteins of candidate p53 target genes found in the *in-silico* study (red and blue), together with p53 (yellow) and 10 most-related proteins (grey) from STRING-db, organized into a network by common properties and interactions. The 16 proteins from our study that are also part of well-connected networks are shown in blue. See S1 File for experimental details.
(TIFF)

S1 File Supplementary Methods. (DOCX)

S1 Table Sequences of DNA oligonucleotides, DNA plasmids and primers for ChIP and qRT-PCR, separate file. (XLSX)

S2 Table Tabulated positions of identified p53CON and longest T.A.*T* triplex sequences relative to the transcription start site of the given RefSeq transcript. Positions of lower stringency p53CON sequences with 2 mismatches are shown in parentheses. Genome coordinates refer to human genome sequence hg38 annotation.
(XLSX)

S3 Table Verification of candidate p53 target genes. *In-silico* candidate gene screening of publicly available microarray and sequencing datasets and summarization of results of verification by RT-qPCR. See S1 File for experimental details.
(XLSX)
