# Supplementary figures and images for: p53 Specifically Binds Triplex DNA In Vitro and in Cells
Source: PLoS One. 2016 Dec 1;11(12):e0167439. doi: 10.1371/journal.pone.0167439 (PMC5131957; doi:10.1371/journal.pone.0167439)

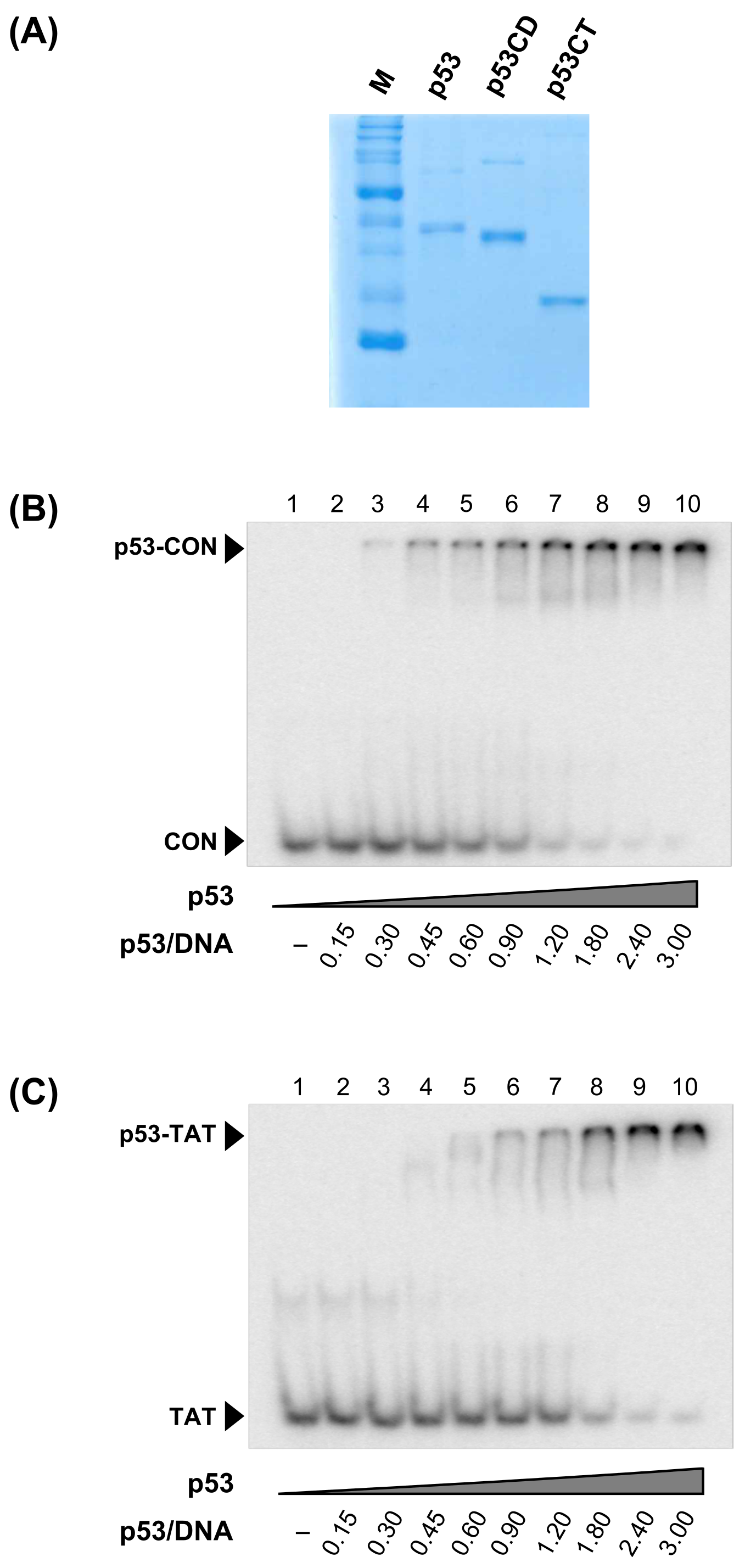

Supplement: S1 Fig — (A) SDS-PAGE analysis of p53 proteins used in the study. The purity and appropriate size of each proteins was analyzed by Coomassie blue staining of 12.5% SDS-PAGE gel. (B) Full length p53 was bound to 1 pmol of 32P-labeled 50-mer oligonucleotides represented by p53 nonspecific dsDNA (NON, lanes 1–5), p53 specific dsDNA with CON (CON, lanes 6–10) and triplex (dT)50.(dA)50.(dT)50 (TAT, lanes 11–17) in the presence of DNA competitor (linear plasmid pBSK/SmaI, 50 ng). The reactions were separated on 4% 0.5× TBM (2 mM MgCl2) polyacrylamide gel (PAGE), 3h. Radioactively labeled DNA was detected by autoradiography. B,C) Full length p53 was bound to 1 pmol of 32P-labeled 50-mer oligonucleotides represented by p53 specific dsDNA with CON (CON, B) and triplex (dT)50.(dA)50.(dT)50 (TAT, C) in the presence of DNA competitor (linear plasmid pBSK/SmaI, 20 ng). The reactions were separated on 5% 0.5× TBM (2 mM MgCl2) PAGE, 1 h. Radiolabeled DNA was detected by autoradiography. (TIFF) [file pone.0167439.s001.tiff]

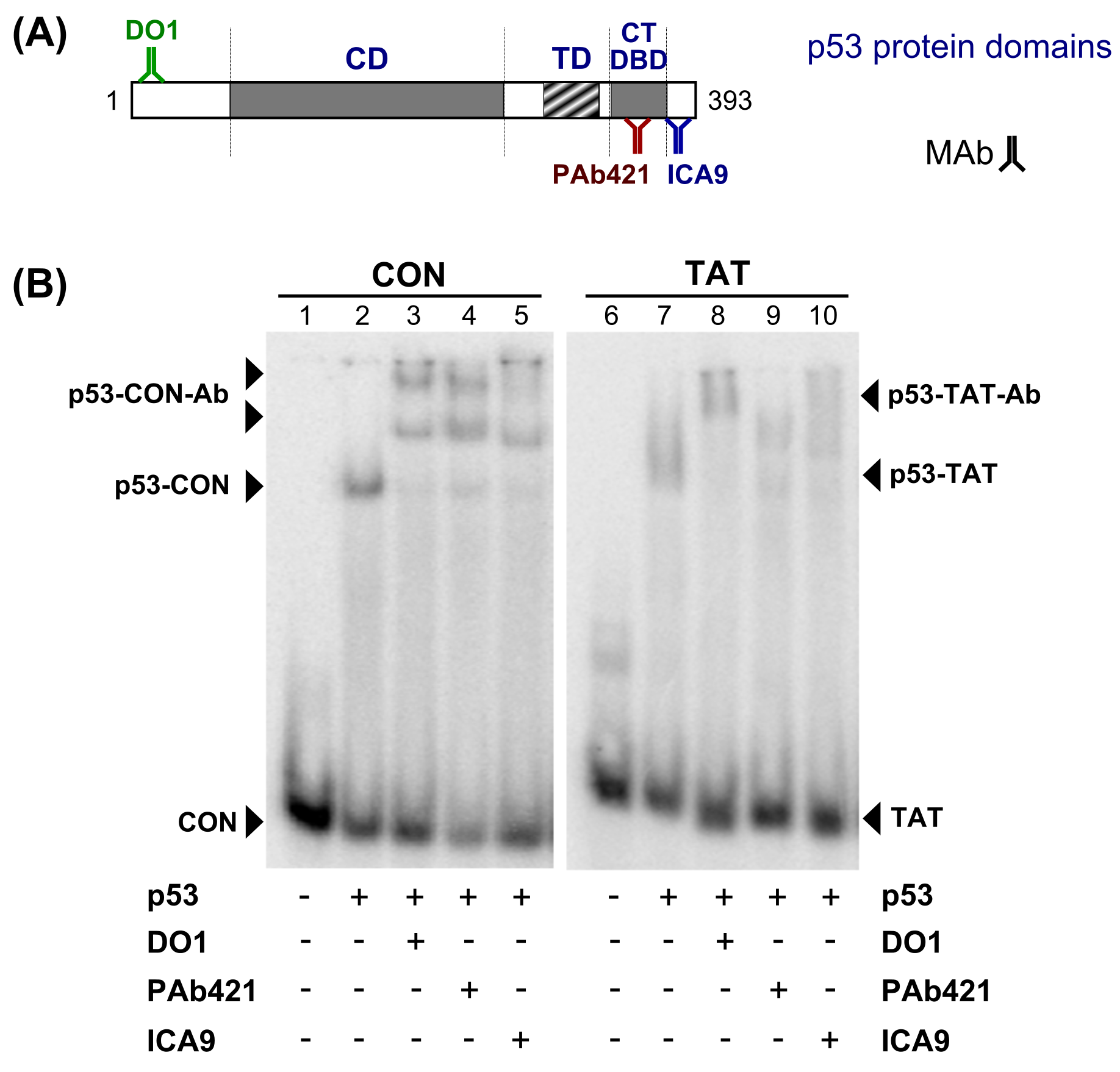

Supplement: S2 Fig — (A) Scheme of p53 used in this study, shown as boxes below the map of p53 domains. The evolutionarily conserved domains are indicated: core DNA binding domain (CD; aa ~100–300), tetramerization domain (TD; aa 325–356) and basic C-terminal DNA binding domain (CTDBD; aa 363–382) and location of p53 antibodies PA421, ICA9 and DO1 used in our study. (B) Effect of C-terminal modifications of p53 protein by Ab on T.A.T recognition. The antibodies (DO1, PAb421 and ICA9; 1.5 μg) were bound to p53 (300 ng) in Ab/p53 molar ratio 2/1 at RT for 15 min. Then 1 pmol of 32P-labeled 50-mer oligonucleotides represented by p53 specific dsDNA with p53CON (CON, lanes 1–5) and triplex (dT)50.(dA)50.(dT)50 (TAT, lanes 6–10) were added and mixtures were incubated at 4°C for 20 min. The reactions were separated on 4% 0.5× TBM (2 mM MgCl2) PAGE at 4°C. Radioactively labeled DNA was detected by autoradiography. Mouse monoclonal anti-p53 antibodies (mAb) (DO1 (aa 20–25), Bp53 10.1 (aa 375–379), PAb421 (aa 371–380) and ICA9 (aa 388–393)) and anti-GST Ab (G1160, Sigma) were used. (TIFF) [file pone.0167439.s002.tiff]

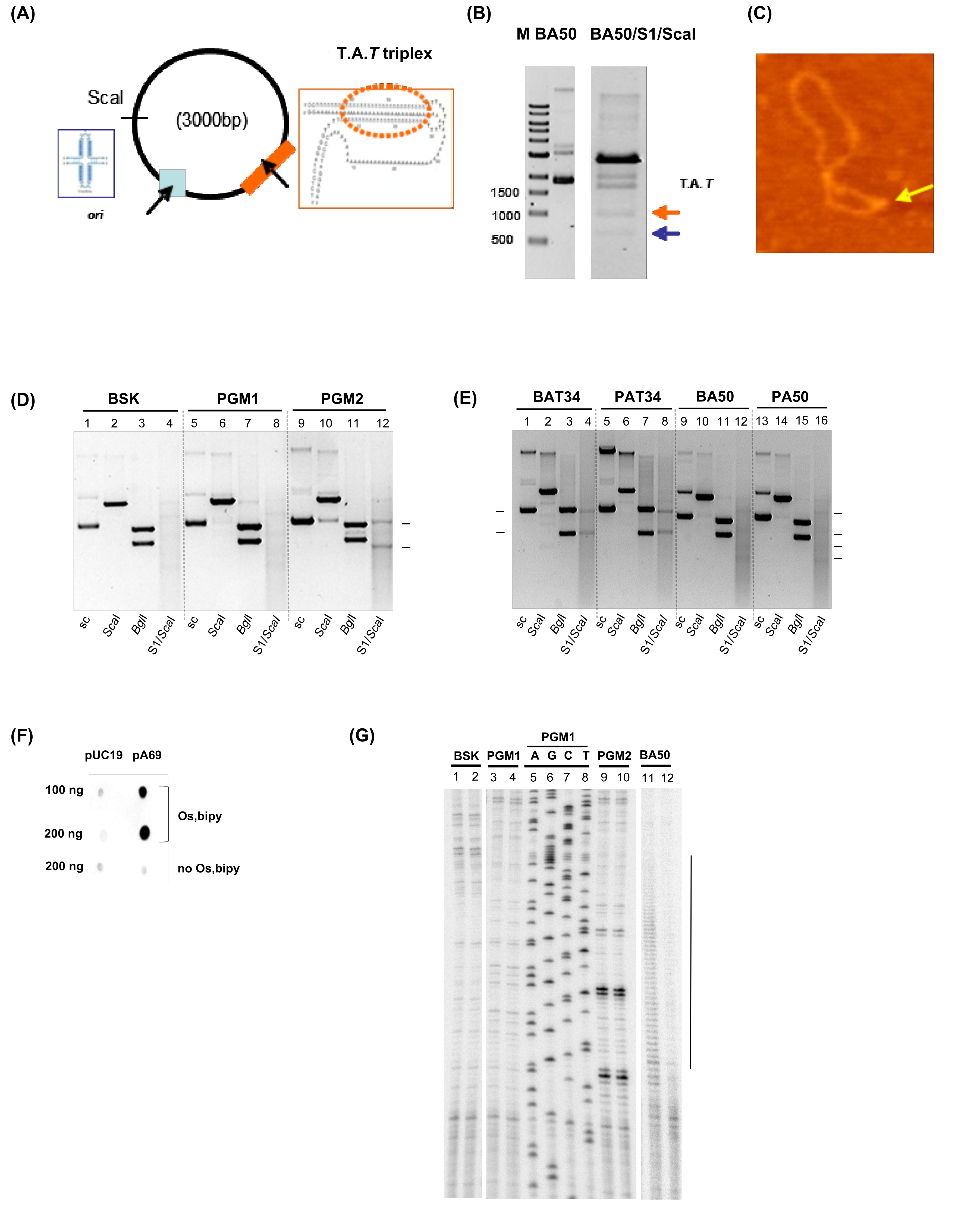

Supplement: S3 Fig — (A,B,D,E) Scheme of non-B DNA structures detection by S1 nuclease treatment described in [30]. scDNAs were treated with S1 nuclease followed by ScaI digestion. Detection of two fragments indicates one major non-B DNA structure (cruciform or triplex) formation in the polycloning site in the case of pBA50 (A), pPGM2 (D, lane 12), pBAT34 (E, lane 4), and pAT34 (E, lane 8). But also pPGM1 (D, lane 8), pBA50 (B; E, lane 12), pPA50 (E, lane 16) and pBSK (D, lane 4) were sensitive to S1 nuclease treatment; two pairs of fragments (black lines) were detected, indicating that all plasmids can form non-B DNA structures with unpaired bases. (C) AFM visualization of intramolecular triplex in pBA50, conditions as described in Fig 2. (F) Detection of non-B DNA modified with OsO4-bipy by dot blot on nitrocellulose membrane with specific antibody against OsO4-bipy-DNA adduct as described in [48]. pUC19 (vector only) and pA69 were modified by condition described in [48]; (G) Detection of non-B DNA in plasmid DNA pre-incubated in 20 mM TrisHCl pH8, 2mM MgCl2 without/with 100 mM NaCl by OsO4-bipy modification followed by primer extension analysis of pBSK (1,2), PGM1 (3,4), PGM2 (9,10), pBA50 (11,12) plasmid DNA, conditions described in [47]. Primer extension from T7 primer was used. See S1 File for experimental details. (TIFF) [file pone.0167439.s003.tiff]

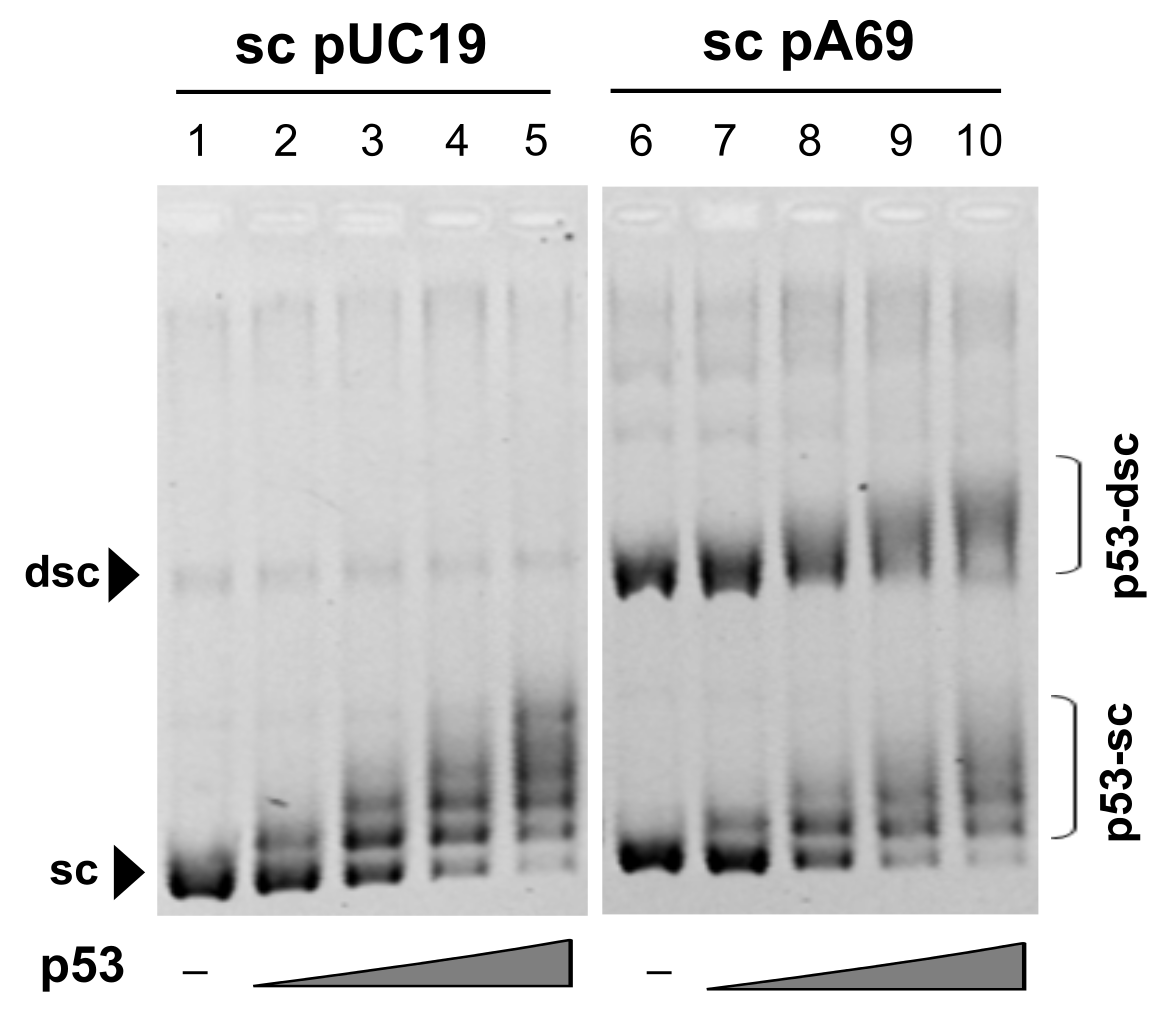

Supplement: S4 Fig — Binding of p53 protein to pUC19 and pA69 detected by EMSA in agarose gel. p53 protein was bound to scDNA (pUC19, 200 ng, lanes 1–5) and scDNA with (dA)69(dT)69 (pA69, 200 ng, 6–10) in p53/DNA molar ratios 1–5 at 25°C, EMSA was performed at 4°C. (TIFF) [file pone.0167439.s004.tiff]

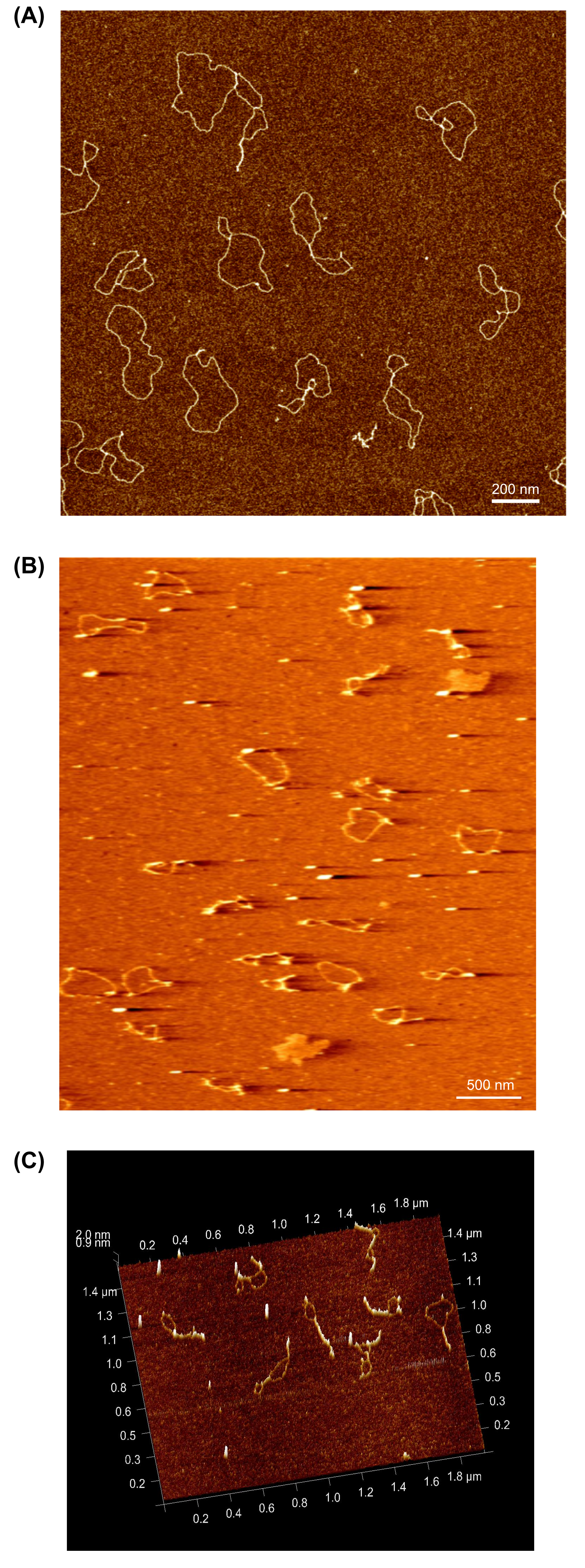

Supplement: S5 Fig — (A) AFM image of scBA50 plasmid mounted in the presence of 5 mM MgCl2. Scale bar represents 200 nm. (B) Image of pA69 complexes with p53, proteins were incubated with DNA in molar ratio 5/1 in DNA binding buffer and then loaded on mica surface in the presence of 5 mM MgCl2. Scale bar represents 500 nm. (C) pA69 plasmid with p53 proteins in 3D projection. (TIFF) [file pone.0167439.s005.tiff]

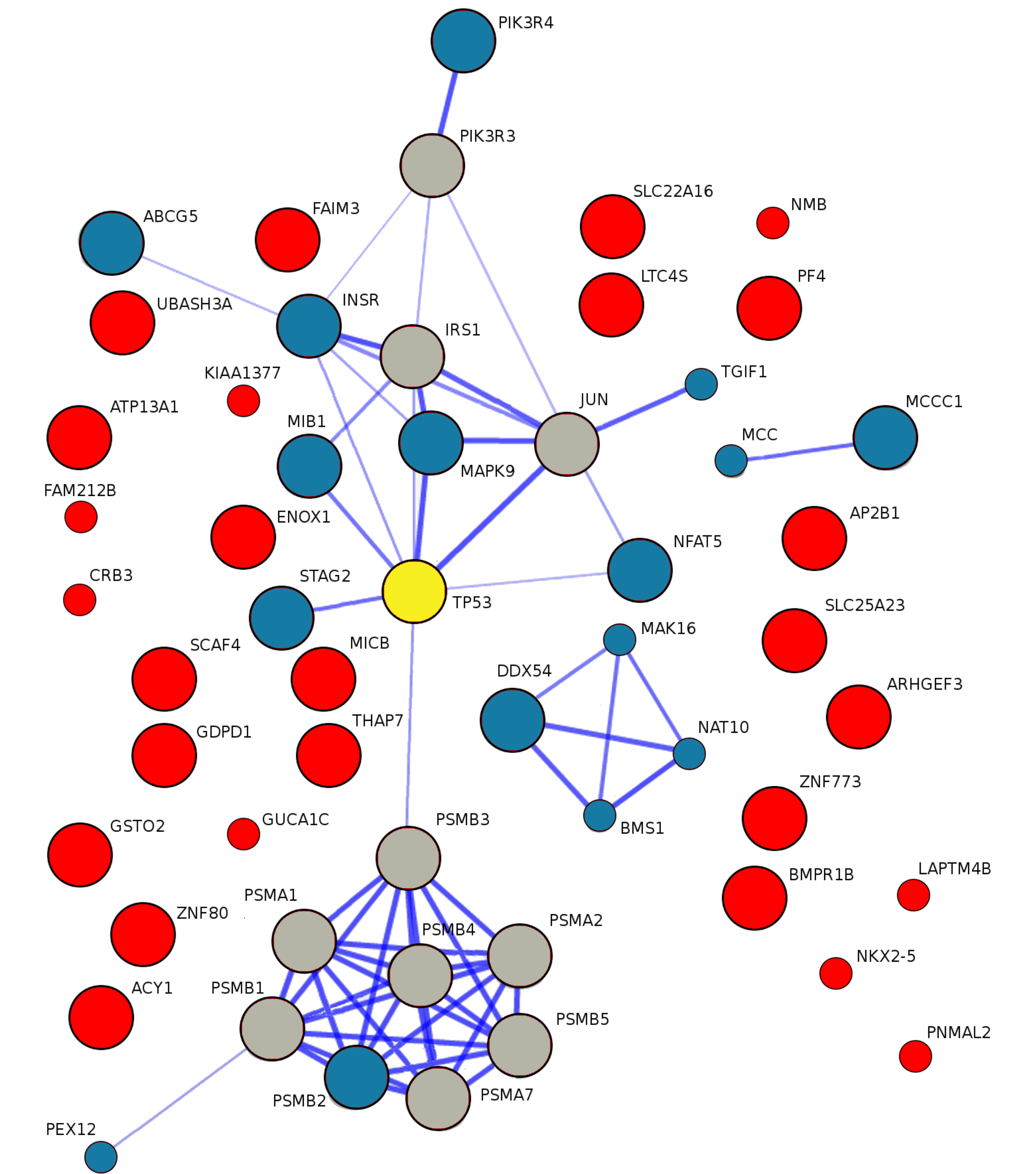

Supplement: S6 Fig — The 43 highest-scoring proteins of candidate p53 target genes found in the in-silico study (red and blue), together with p53 (yellow) and 10 most-related proteins (grey) from STRING-db, organized into a network by common properties and interactions. The 16 proteins from our study that are also part of well-connected networks are shown in blue. See S1 File for experimental details. (TIFF) [file pone.0167439.s006.tiff]
