# Supplementary material for: p53 Specifically Binds Triplex DNA In Vitro and in Cells
Source: PLoS One. 2016 Dec 1;11(12):e0167439. doi: 10.1371/journal.pone.0167439 (PMC5131957; doi:10.1371/journal.pone.0167439)
Supplement: S1 File — (DOCX) [file pone.0167439.s007.docx]

## S1 File. Supplementary Methods

**Structure-specific binding of tumor suppressor p53 protein to triplex DNA *in vitro* and in cells**

**Marie Brázdová^1*^, Vlastimil Tichý^1^, Robert Helma^1^, Pavla Bažantová^1^, Alena Polášková^1^, Aneta Krejčí^2^, Marek Petr^1^, Lucie Navrátilová^1^, Olga Tichá^1^, Karel Nejedlý^1^, Martin L. Bennink^3^, Vinod Subramaniam^3^, Zuzana Bábková^2^, Tomáš Martínek^4^, Matej Lexa^5^ and Matej Adámik^1^**

^1^Department of Biophysical Chemistry and Molecular Oncology, Institute of Biophysics, Academy of Sciences of the Czech Republic v.v.i., Královopolská 135, Brno, 61265, Czech Republic

^2^[Department of Molecular Biology and Pharmaceutical Biotechnology, Faculty of Pharmacy, University of Veterinary and Pharmaceutical Sciences, Palackého 1/3, 61242 Brno, Czech Republic](http://www.muni.cz/sci/314010/people)

^3^Biophysical Engineering Group, Faculty of Science and Technology, University of Twente, Enschede, 7500AE, The Netherlands

^4^Department of Computer Systems, Faculty of Information Technology, Brno University of Technology, Brno, 612 66, Czech Republic

^5^Department of Information Technologies, Faculty of Informatics, Masaryk University, Brno, 60200, Czech Republic

* To whom correspondence should be addressed. Tel: +420 541 517 176; Fax: +420 541 211 293; Email: maruska@ibp.cz

**Methods**

**Nuclease S1 and restriction enzyme assays.**

Supercoiled plasmid constructs (2 µg) after preincubation in triplex forming buffer were digested with 4U of S1 nuclease (Promega) in 18 μl of S1 buffer for 20 min at 37 °C. The S1 nuclease reaction was quenched with stop buffer (500 mM Tris HCl pH 8, 125 mM EDTA), followed by heating for 10 min at 70 °C. The resulting S1-digested plasmids were recovered by precipitation and digested further with *Sca*I. The products were resolved by agarose gel electrophoresis. For restriction enzyme assays, supercoiled plasmid DNA was digested with *Sca*I (Takara) for 60 min at 37 °C. ScDNAs (pPGM1, pPGM2, pBA50, pPA50, pBSK) were treated with S1 nuclease followed *Sca*I restrictase digestion [[1](#_ENREF_1), [2](#_ENREF_2)]. In case of pPGM2 (S3D Fig, lane 12), pBA34 (S3E Fig, lane 4), pPAT34 (S3E Fig, lane 8), detection of two fragments (about 1100 and 1800 bp) indicates DNA cruciform formation. pBA50 (S3E Fig, lane 12; S3B Fig), pPA50 (S3E Fig, lane 12) were sensitive to S1 nuclease treatment; two pairs of fragments were detected, indicating that pBA50 and pPA50 bases can form some non-B DNA structures with unpaired bases, linDNA was about 2960 bp long.

## Analyses of DNA structure by chemical probing with OsO_4_-bipy and primer extension

Purified scDNA were analyzed chemically with OsO4-bipy. scDNA plasmids (2 µg) were preincubated in A buffer (triplex forming buffer, 20 mM TrisHCl pH 8, 100 mM NaCl, 2 mM MgCl_2_, 0.1 mM EDTA) or B buffer (20 mM TrisHCl pH 8, 0.1 mM EDTA) for 30 min at 37 °C. DNA modifications by OsO4-bipy were performed using radioactively labeled DNA (20 kcpm), which was equilibrated in 50 μl of DNA binding buffer (without DTT) for 30 min at RT followed by addition of 50 μl OsO4-bipy solution (4 mM OsO_4_ and 4 mM 2,2’-bipyridine in B buffer). After 15 min incubation at RT reactions were stopped by phenol-chloroform extraction and after ethanol precipitation the samples were prepared for primer extension.

## Primer extension was performed according to [[3](#_ENREF_3)] with some modifications. 15 to 20 pmol of the primer were labeled in a final volume of 10 μl by 16 pmol of [γ-P32]ATP and 5 units of T4 polynucleotide kinase for 20 min at 37 °C. After addition of another 5 units of the enzyme, the incubation continued for another 20 min. The enzyme was thermally inactivated (65 °C, 20 min) and the labeled primer was devoid of unincorporated ATP and other small molecules by repeated centrifugal gel filtration (Centri.Spin-10; Princeton Separations) according to the manufacturer's instructions. The radioactivity of the lyophilized eluate was measured and set to 0.40–0.45×10^6^ cpm/μl after dissolution. Primer extension with the linearized plasmid followed the protocol referred to above. Briefly, sodium hydroxide denatured template (about 0.14 pmol) and the primer (about 6×10^5^ cpm) were neutralized, hybridized at 56 or 57 °C (upper and bottom strand primer, respectively) for 3 min, and cooled. After the addition of deoxynucleoside triphosphates and the Klenow fragment (1 unit), primer extension proceeded for 10 min at 50 °C. The EDTA terminated samples were precipitated, washed, and dried. The radioactivity of the samples was measured and dissolved to a concentration of 1×10^5^ cpm/μl in a sequencing gel-loading solution.

**Non-B DNA detection with dot blot and antibody recognizes DNA modified with Os,bipy**

Modification of scDNA with OsO4-bipy was done as described above, DNA after cleaning with phenol-chloroform extraction and after ethanol precipitation the samples were diluted in TE buffer to final concertation 100 ng/1µl and 1 and 2 µl samples (pUC19 and pA69) were loaded on nitrocellulose membrane together with no modified controls. DNA-OsO4-bipy adducts were detected by a monoclonal antibody OsBP7H8 (1:10 supernatant) against by standard immunodetection described in [[4](#_ENREF_4)]. OsBP7H8 was produced in our laboratory [[5](#_ENREF_5)].

***In-silico* candidate gene screening**

Candidate gene transcription was checked in publicly available microarray and sequencing datasets from experiments involving p53-transformed cells originally lacking active p53 or experiments were p53 was activated by nutlin-3, 5-fluoruracil or doxorubicin (SRP043273 [[6](#_ENREF_6)], SRP022871 [[7](#_ENREF_7)], E-GEOD-30753 [[8](#_ENREF_8)], E-GEOD-50650 [[9](#_ENREF_9)], E-GEOD-8660 [[10](#_ENREF_10)], E-MEXP-2556 [[11](#_ENREF_11)], [[12](#_ENREF_12)]). We obtained expression data from tables available from the iRAP pipeline [[13](#_ENREF_13)], deposited by authors to Array Express [[14](#_ENREF_14)] or calculated from the available data using the ArrayExpress R/Bioconductor package [[15](#_ENREF_15)]. Raw expression values were normalized relative to GAPDH housekeeping gene and averaged, where replicates were available.

**References:**

1. Brazdova M, Navratilova L, Tichy V, Nemcova K, Lexa M, Hrstka R, et al. Preferential binding of hot spot mutant p53 proteins to supercoiled DNA in vitro and in cells. PLoS One. 2013;8(3):e59567.

2. Palecek E, Brazda V, Jagelska E, Pecinka P, Karlovska L, Brazdova M. Enhancement of p53 sequence-specific binding by DNA supercoiling. Oncogene. 2004;23(12):2119-27.

3. Nejedly K, Chladkova J, Kypr J. Photochemical probing of the B--a conformational transition in a linearized pUC19 DNA and its polylinker region. Biophys Chem. 2007;125(1):237-46.

4. Brazdova M, Palecek J, Cherny DI, Billova S, Fojta M, Pecinka P, et al. Role of tumor suppressor p53 domains in selective binding to supercoiled DNA. Nucleic Acids Res. 2002;30(22):4966-74.

5. Buzek J, Kuderova A, Pexa T, Stankova V, Lauerova L, Palecek E. Monoclonal antibody against DNA adducts with osmium structural probes. J Biomol Struct Dyn. 1999;17(1):41-50.

6. Sanchez Y, Segura V, Marin-Bejar O, Athie A, Marchese FP, Gonzalez J, et al. Genome-wide analysis of the human p53 transcriptional network unveils a lncRNA tumour suppressor signature. Nat Commun. 2014;5:5812.

7. Janky R, Verfaillie A, Imrichova H, Van de Sande B, Standaert L, Christiaens V, et al. iRegulon: from a gene list to a gene regulatory network using large motif and track collections. PLoS computational biology. 2014;10(7):e1003731.

8. Kracikova M, Akiri G, George A, Sachidanandam R, Aaronson SA. A threshold mechanism mediates p53 cell fate decision between growth arrest and apoptosis. Cell death and differentiation. 2013;20(4):576-88.

9. Bisio A, De Sanctis V, Del Vescovo V, Denti MA, Jegga AG, Inga A, et al. Identification of new p53 target microRNAs by bioinformatics and functional analysis. BMC Cancer. 2013;13:552.

10. Sauer M, Bretz AC, Beinoraviciute-Kellner R, Beitzinger M, Burek C, Rosenwald A, et al. C-terminal diversity within the p53 family accounts for differences in DNA binding and transcriptional activity. Nucleic acids research. 2008;36(6):1900-12.

11. Kudoh T, Kimura J, Lu ZG, Miki Y, Yoshida K. D4S234E, a novel p53-responsive gene, induces apoptosis in response to DNA damage. Exp Cell Res. 2010;316(17):2849-58.

12. Allen MA, Andrysik Z, Dengler VL, Mellert HS, Guarnieri A, Freeman JA, et al. Global analysis of p53-regulated transcription identifies its direct targets and unexpected regulatory mechanisms. Elife. 2014;3:e02200.

13. Fonseca NA, Marioni J, Brazma A. RNA-Seq gene profiling--a systematic empirical comparison. PLoS One. 2014;9(9):e107026.

14. Kolesnikov N, Hastings E, Keays M, Melnichuk O, Tang YA, Williams E, et al. ArrayExpress update--simplifying data submissions. Nucleic acids research. 2015;43(Database issue):D1113-6.

15. Kauffmann A, Rayner TF, Parkinson H, Kapushesky M, Lukk M, Brazma A, et al. Importing ArrayExpress datasets into R/Bioconductor. Bioinformatics (Oxford, England). 2009;25(16):2092-4.
